# Supplementary material for: SEMORE: SEgmentation and MORphological fingErprinting by machine learning automates super-resolution data analysis
Source: Nat Commun. 2024 Feb 26;15:1763. doi: 10.1038/s41467-024-46106-0 (PMC10897458; doi:10.1038/s41467-024-46106-0)
Supplement: Supplementary file 1 — Supplementary Information [file 41467_2024_46106_MOESM1_ESM.pdf]

Supplementary Information

SEMORE: SEgmentation and MORphological fingErprinting by machine learning automates super-resolution data analysis

Steen W. B. Bender[1,2,3], Marcus W. Dreisler[1,2,3], Min Zhang[1,2,3], Jacob Kæstel-Hansen*[1,2,3] & Nikos S. Hatzakis*[1,2,3,4]

[1]Department of Chemistry, University of Copenhagen, Denmark, [2]Center for 4D cellular dynamics, University of Copenhagen, Denmark, [3]Novo Nordisk Center for Optimised Oligo Escape and Control of Disease, University of Copenhagen, Denmark, [4]Novo Nordisk Center for Protein Research, University of Copenhagen, Denmark,.

*Correspondence: e-mail: jkh@chem.ku.dk e-mail: hatzakis@chem.ku.dk

Supplementary Tables 2
Supplementary Table 1: Description of each morphology fingerprint feature. 2
Supplementary Figures 3
Supplementary Fig. 1: Complete pipeline and resulting effect of introduced smart density filter. 3
Supplementary Fig. 2: SEMORE out-of-box noise stress test of non-overlapping highly diverse aggregates. 4
Supplementary Fig. 3: Benchmarking SEMORE on for heterogeneous, non-uniform noise. 5
Supplementary Fig. 4: Comparison of operational performance of segmentation module in stress test containing simulated overlapping protein aggregates where temporal information is included.6
Supplementary Fig. 5: Visual representation of SEMORE's ability to capture morphological growth in time. 7
Supplementary Fig. 6: Deconvolution of temporal refinement on real and simulated data. 8
Supplementary Fig. 7: Evaluation of SEMORE' segmentation and structural information extraction on simulated small tetramer assemblies. 9
Supplementary Fig. 8: SEMORE classification performance for small, sparse clusters of diverse morphologies. 10
Supplementary Fig. 9: Effect of blinking on SEMORE's morphological fingerprinting and characterization of morphological classes. 12
Supplementary Fig. 10: Demonstration SEMORE clustering of morphology shrinkage. 13
Supplementary Fig. 11: Classification capabilities of unsupervised morphology fingerprinting, in high-density regions of diverse aggregate morphologies for SMLM without temporal features. 14
Supplementary Fig. 12: Resulting UMAP from SEMORE clustering applied on the simulated structures with the smart density filtering. 15
Supplementary Fig. 13: Fibril branch classification performance through feature-class specific investigation. 16
Supplementary Fig. 14: Simulation type-specific UMAP investigation based on the circularity feature class. 17
Supplementary Fig. 15: Depiction of recurrent SEMORE fingerprinting for dynamic morphology variation of protein clusters. 18
Supplementary Fig. 16: UMAP feature importances. 19
Supplementary Fig. 17 dSTORM data from Nieves et. al1 clustered and quantified by SEMORE. 20
Supplementary Fig. 18: Evaluation of SEMORE on temporarily resolved Sx1a-mEos2 sptPALM data2. 21
Supplementary Fig. 19: Evaluation of SEMORE on temporarily resolved live-cell PALM data of ryanodine receptors (RyRs)3. 22
Supplementary Fig. 20: Demonstration and visualization of structure-polygon for size estimation. 23
Supplementary Fig. 21: Pipeline for morphology edge estimation. 24
Supplementary Fig. 22: All the used spatial features distributions from nuclear pore complex treatment from the NPC-A647 dataset4. 25
Supplementary Fig. 23: Morphology fingerprint feature distributions for anisotropic and isotropic growth type aggregates from the insulin aggregation studies by REPLOM5. 26
Supplementary Fig. 24: The 27 aggregates with the biggest and smallest areas of the anisotropic classified structures from the insulin aggregation studies by REPLOM5. 27
Supplementary Fig. 25: The 27 aggregates with the biggest and smallest areas of the isotropic classified structures from the insulin aggregation studies by REPLOM5. 28
Supplementary Fig. 26: SEMORE's Morphological Fingerprint captures gradual transitions in morphology. 29

Supplementary Tables

Supplementary Table 1: Description of each morphology fingerprint feature.

#	Feature	Sub-set	Description
1	x ratio	Symmetry	Ratio of points between the left and right side of the y-axis
2	y ratio	Symmetry	Ratio of points between above and below the x-axis
3	ratio sym	Symmetry	x ratio * y ratio
4	x dist ratio	Symmetry	Ratio of longest absolute distance compared to total span (x-axis)
5	y dist ratio	Symmetry	Ratio of longest absolute distance compared to total span (y-axis)
6	Pearson	Symmetry	Pearson coefficient.
7	Spearman	Symmetry	Spearman coefficient
8	Area	Geometric	Area estimation found through accumulation of triangle areas.
9	Density	Geometric	Points in aggregate divided with area.
10	Cut distance	Geometric	Longest allowed distance for pairwise connection
11	Mean k	Graph network	Average amount of neighbors/connection
12	Median k	Graph network	Median of neighbors/connection
13	K max	Graph network	Max of neighbors/connection
14	L_s_d	Graph network	Distance between most separated points
15	L_s_path	Graph network	Distance along minimum spanning tree between most separated points
16	L_s_step	Graph network	Number of bridges used in L_s_path
17	L_s_mean	Graph network	Average distance connection in L_s_path
18	L_s_median	Graph network	Median of distance connection in L_s_path
19	L_l_max	Graph network	Max distance connection in L_s_path
20	L_s_effectiveness	Graph network	L_s_d divided with L_s_step
21	L_s_ratio	Graph network	Ratio between the L_s_d and L_s_path
22	L_l_path	Graph network	Longest possible one-way road through the minimum spanning tree
23	L_l_d	Graph network	Direct distance between start and end points in L l path
24	L_l_step	Graph network	Same as 16 now with L_l_path
25	L_l_mean	Graph network	Same as 17 now with L_l_path
26	L_l_median	Graph network	Same as 18 now with L_l_path
27	L_l_max	Graph network	Same as 19 now with L_l_path
28	L_l_effectiveness	Graph network	Same as 20 now with L_l_path
29	L_l_ratio	Graph network	Same as 21 now with L_l_path
30	Ls_ll_d ratio	Graph network	Ratio between L_s_d and L_l_d
31	Ls_ll_path ratio	Graph network	Ratio between L_s_path and L_l_path
31+N	Mu 1-N	Graph network	Mean of N fitted Gaussians over connections (k)
31+2N	Sig 1-N	Graph network	Standard deviation of N fitted Gaussians over connection (k)
31+3N	W 1-N	Graph network	Weights of N fitted Gaussians over connection (k)
32+3N	State diff	Graph network	Average difference between Gaussians
33+3N	Variance	Circularity	Variance of distance from the core to morphology edge
34+3N	Circularity	Circularity	$4 \cdot \pi \cdot area \ / \ circumfrence^2$
35+3N	Convexity	Circularity	The area of convex hull dived with area of contour line
36+3N	Circ inertia	Circularity	Assuming ellipse, ratio between vertex
37+3N	Sph value	Circularity	combination of the 4 circularity features E[(1 −V ar) + Circ + Conv + Inert]

Supplementary Figures

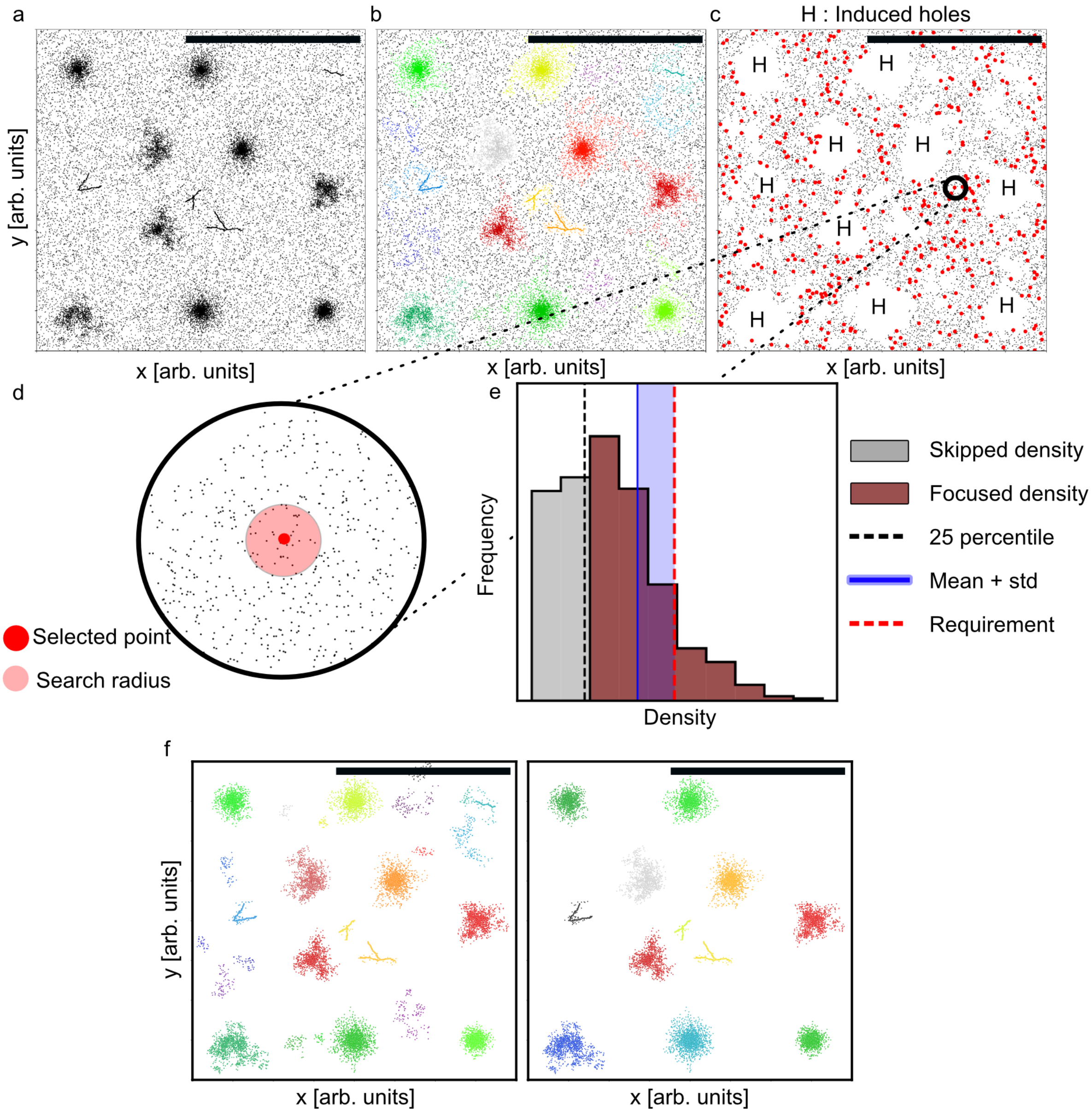

Supplementary Fig. 1: Complete pipeline and resulting effect of introduced smart density filter.
a, Raw simulated data with an experimental relevant noise ratio. **b,** the initial clustering with noise in black and the high-density areas in colors. **c,** The initial found noise, with the high-density areas removed, resulting in multiple holes (denoted "H"). 500 noise points (red) are randomly selected for noise density estimation. **d,** A zoom-in of a selected noise point. For each selected point a search radius of 0.03 (in standardized space) is drawn from which the density is calculated. **e,** The accumulated densities in a histogram. Due to the holes present in **c,** some selected points may experience sparse densities, to account for this, a 25-percentile mask is applied (grey) to avoid any underestimation. This results in focused densities (red-brown) from which a minimum density requirement is then calculated as the mean + the standard deviation. **f,** Comparison visualization, the presented data set treated through SEMORE without the smart density filtering, clearly extracting multiple non-aggregate structures, while when applied completely removing these structures, drastically lowering the overall false positive rate. Black scale bar is 40000 arb.u.

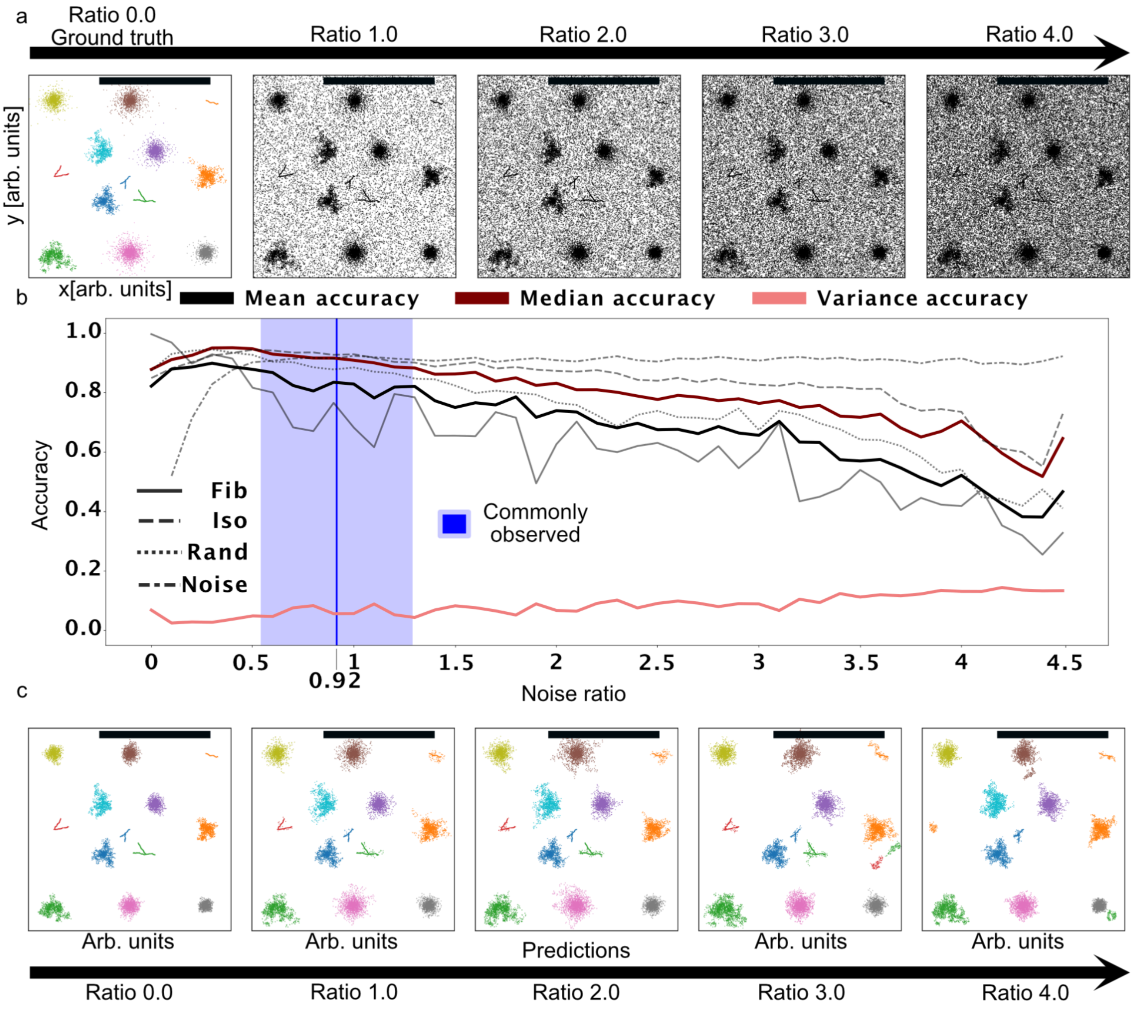

Supplementary Fig. 2: SEMORE out-of-box noise stress test of non-overlapping highly diverse aggregates.

a, 5 experiments were simulated without noise, each containing 13 aggregates of randomly selected simulation types. To ensure minimal overlapping, each aggregate was kept in the same grid throughout the 5 experiments. The noise was incremented for each experiment with a 0.1 ratio (N_noise / N_label) up to a 4.5 noise ratio. **b,** SEMORE accuracy (see Methods) against noise ratio increase. Both mean (black) and median (dark red) start with a positive slope and reach their highest performance at 90% and 95% respectively for a noise ratio of ~0.4, which corresponds to the general assumption of an unavoidable noise present in the training sets (grey dash-dot). Further noise ratio increases results in a linear decrease in the accuracy. Fibril (gray solid) displays the steepest decline, due to its smaller spatial occupation and density compared to isotropic (gray dashed) and random (gray short dashed). At an unreasonably high noise ratio of noise 4.5, the mean and median were 38% and 52% respectively with a variance of around 15%. The biologically relevant and experimentally recorded noise of 0.92 is displayed with a blue line with a spread of 0.37 shown as blue transparency on both sides. **c,** Predicted classification labels for the noise ratios. SEMORE generally separates noise from labels for experimentally relevant noise (<1). At higher noise density, the predictions include more false positive locations and whole aggregates disappearing. In the experimentally relevant noise range, SEMORE correctly outputs the underlying structures at median accuracy of up to 93%. Black scale bars are 40000 arb.u.

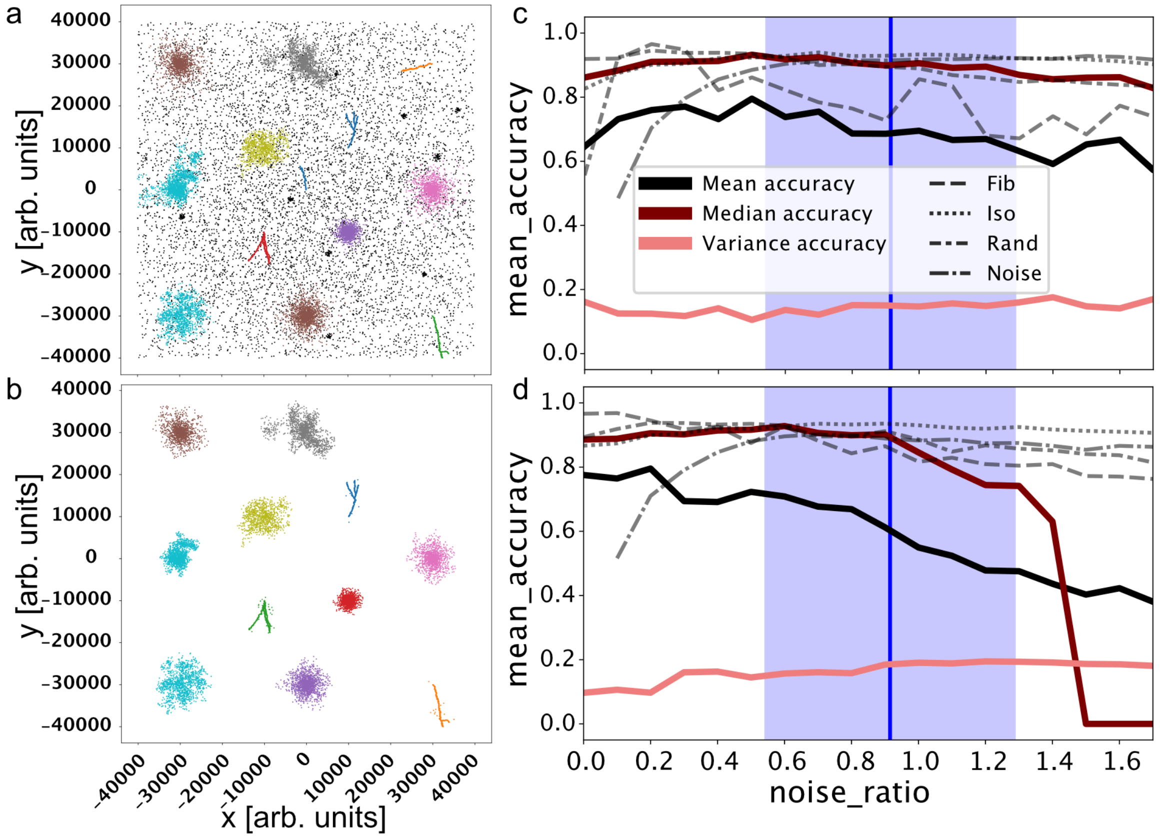

Supplementary Fig. 3: Benchmarking SEMORE on for heterogeneous, non-uniform noise.
a, Simulated data as described and used in SI fig. 2, that includes 5 separate simulations with 13 protein assemblies each, but now exhibiting a heterogeneous noise profile. Heterogeneous noise is generated by introducing 5-25 "noise seeds" each containing 20-50 points have been added to each simulation, additionally, individual Gaussian distributed shifts have been applied to all initial noise points. **b,** we see SEMORE clustering classification performance as out-of-the-box. **c,** Evaluating SEMORE clustering performance versus increasing noise ratio (blue area representing biologically relevant noise-levels estimated from real data and with vertical blue line representing insulin experiments). At biologically relevant noise levels or high noise levels accuracies of SEMORE is observed to be above ~90% with variance accuracy of ~12% from noise ratio ~0.2 and onwards with up to 93% at 0.7 noise ratio reflecting SEMORE's ability to separate non-uniform noise also observed in robust treating of experimental insulin data (see main fig. 4). The unrealistic conditions with extremely low noise ratios or no noise at all are associated with lower accuracies as the smart-density noise filter expects noise and removes true positives if no noise is present, which is easily countered by turning it off. **d,** SEMORE's clustering performance on the same data set without the smart noise filter (blue area representing biologically relevant noise-levels estimated from real data and with vertical blue line representing insulin experiments). SEMORE accuracy is above ~90% from conditions with no noise up to around noise ratios of ~1. Turning off the noise filter results in better performance at extremely and unrealistically low or no noise ratios (zero noise to noise-to-signal ratio of 0.1), but the performance strongly declines starting from around biologically relevant noise levels. For users operating with little or no noise in their experimental system the results indicate the smart filter should be turned off while users operating with realistic noise are recommended to utilize the full SEMORE pipeline.

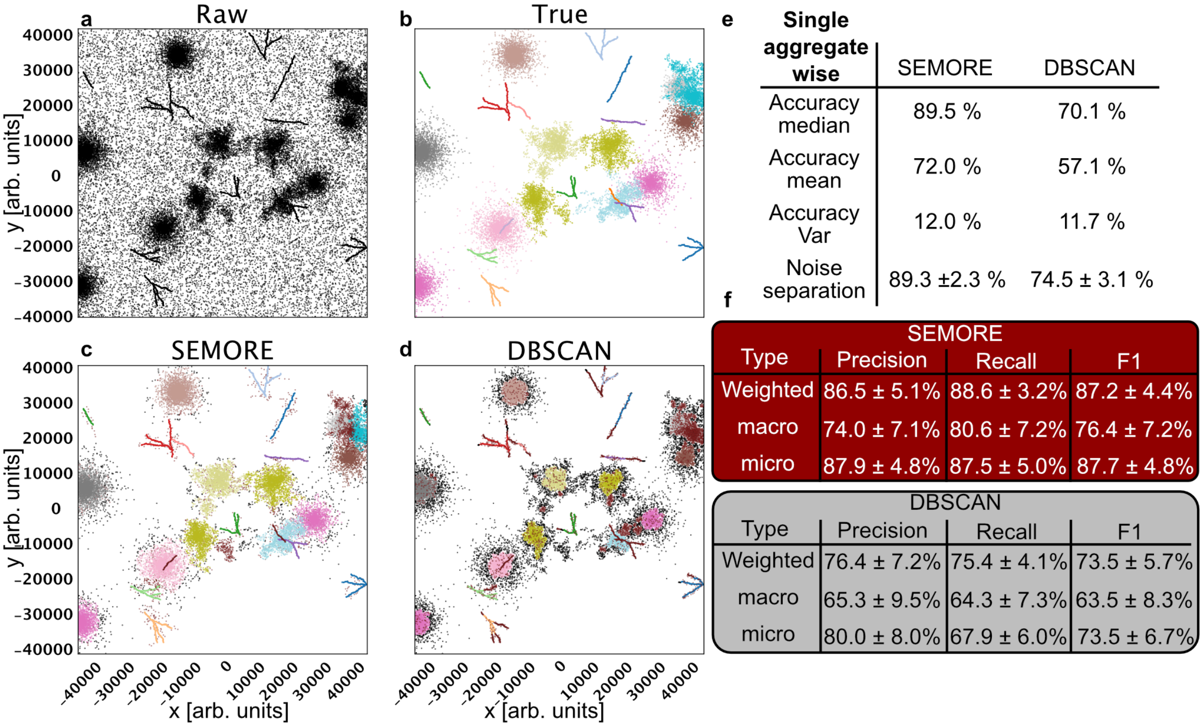

e

Single aggregate wise	SEMORE	DBSCAN
Accuracy median	89.5 %	70.1 %
Accuracy mean	72.0 %	57.1 %
Accuracy Var	12.0 %	11.7 %
Noise separation	89.3 ±2.3 %	74.5 ± 3.1 %

f

SEMORE			
Type	Precision	Recall	F1
Weighted	86.5 ± 5.1%	88.6 ± 3.2%	87.2 ± 4.4%
macro	74.0 ± 7.1%	80.6 ± 7.2%	76.4 ± 7.2%
micro	87.9 ± 4.8%	87.5 ± 5.0%	87.7 ± 4.8%

DBSCAN			
Type	Precision	Recall	F1
Weighted	76.4 ± 7.2%	75.4 ± 4.1%	73.5 ± 5.7%
macro	65.3 ± 9.5%	64.3 ± 7.3%	63.5 ± 8.3%
micro	80.0 ± 8.0%	67.9 ± 6.0%	73.5 ± 6.7%

Supplementary Fig. 4: Comparison of operational performance of segmentation module in stress test containing simulated overlapping protein aggregates where temporal information is included.
a, Representative visualization of the raw data based on simulations containing all three simulation-aggregation types resulting in a highly dense and overlapping aggregate diverse system. **b,** True label illustration without noise. (**c, d)** Segmentation by our method and DBSCAN. Wrongly labelled aggregates are colored brown and wrongly predicted noise is colored black. Both methods' hyperparameters were optimized once for the same simulated experiment and were used for multiple simulations. DBSCAN misses or incorrectly classifies aggregates in proximity and when structures overlap. **e,** Accuracy measure (for 50 experiments containing 25 aggregates each totaling 1250 aggregates) defined as TP/ (TP +FP +FN). As this measure is done for each individual aggregate, the accuracy ranges between 0 and 1 for all structures removing the bias imposed by the large aggregate size variations. SEMORE achieved 89.5% median accuracy, 19.4% higher than DBSCAN with only a 0.3% increase in variance. Furthermore, SEMORE achieves a higher noise separation as well, resulting in both more and cleaner aggregates. **f,** Performance tables for both models with all metrics extracted per experiment. Due to the high diversity of the experiments multiple evaluation approaches are reported: Weighted being evaluation metric per label weighted by TP per label. Macro being the metric for each label unweighted. Micro being total TP, FN and FP combined to a global metric. Errors are standard deviations calculated on an aggregate basis, across 50 simulations. In general, SEMORE outperforms DBSCAN mainly due to its lack of ability to utilize the temporal element, although the temporal dimension was provided to DBSCAN, as well as the addition of density smart filtering.

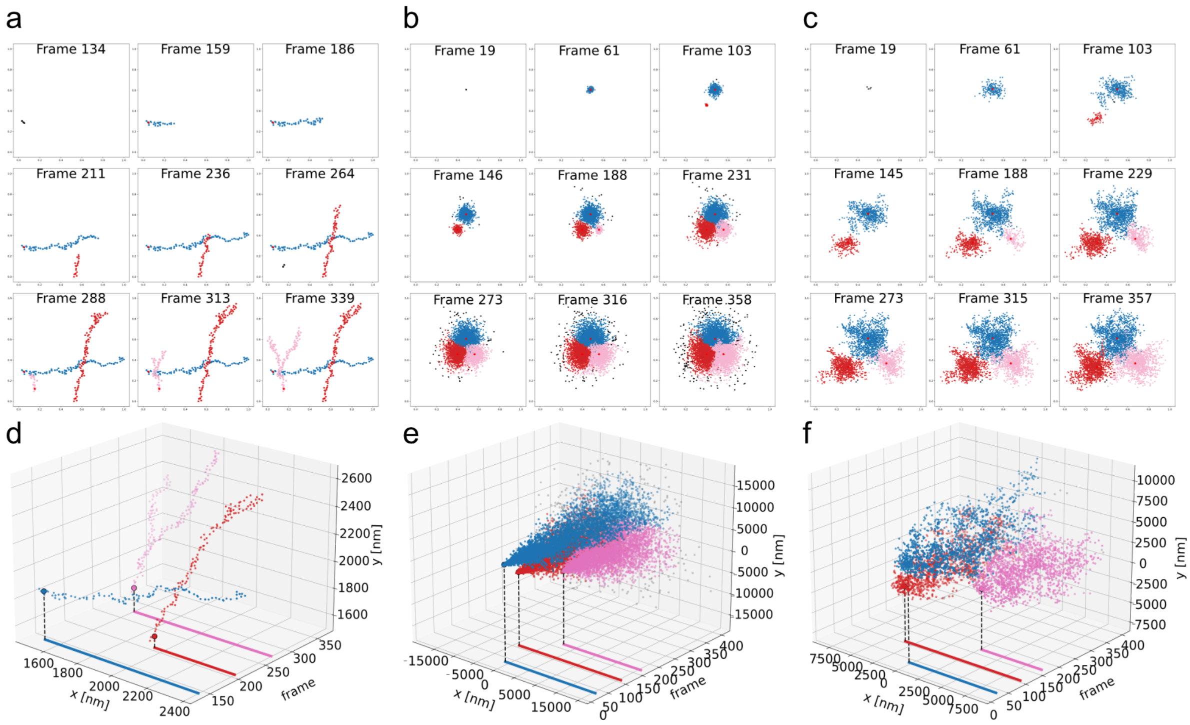

Supplementary Fig. 5: Visual representation of SEMORE's ability to capture morphological growth in time.

(a, b, c) depicts the inherent process of temporal segmentation of assemblies within the clustering module of SEMORE during temporal refinement shown as 9 windows across three morphological classes. **a,** 3 simulated fibrils that spatially overlap during growth are accurately segmented. **b,** 3 simulated isotropic assemblies growing very close to each other accurately extracted and separated by SEMORE. **c,** Simulated growth of 3 sterically-hindered assemblies captured by SEMORE. **(a-c)** showcases the versatility of the general clustering module of SEMORE in capturing temporal evolution of protein assemblies, while general segmentation performance can be seen in main fig 2. The second row **(d-f)**, shows the structures in 3D with the time being the 3rd axis to visualize assembly growth and morphology evolution. A direct depiction of the predicted assembly initiation point is shown as projected lines to the bottom of the plots revealing SEMORE's ability to keep account of various growth onset times. This is shown for Fibril **(d)**, Isotropic **(e)** and sterically hindered growth **(f)** assemblies.

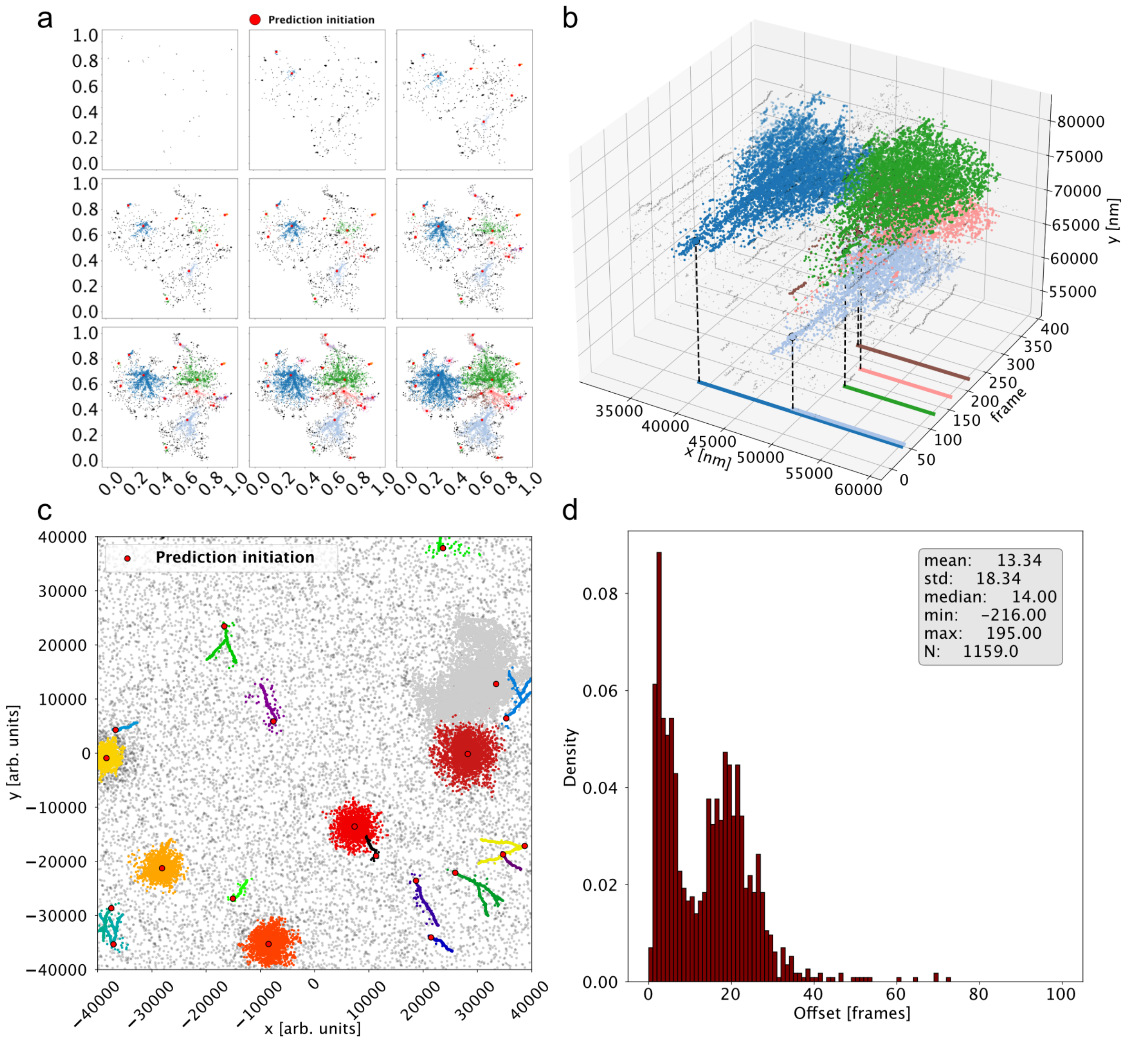

Supplementary Fig. 6: Deconvolution of temporal refinement on real and simulated data.
a, Snapshots of SEMORE's clustering module output at various frames during temporal refinement of found insulin aggregates (also depicted in **Fig. 1C.)** in MinMax transformed space (see Methods). Data displayed in 9 windows equally spaced throughout the 381 frame period, each initially found core point of all dissected aggregates is depicted with a red dot symbolizing the initiation of an aggregate. **b,** The cluster growth is directly visualized in non-transformed space revealing cluster growth in time of **(a)** in a 3D plot after the application of the smart-density filtering (see methods). The cone-like shape of the aggregates in the positive time direction demonstrates SEMORE's ability to capture the growth behaviour of assemblies in experimental data. Each depicted cluster has its predicted initiation point marked with projected lines at the bottom of the plot to highlight the vast difference in the onset times of clusters as revealed by SEMORE. **c,** Simulated data (from SI fig. 3) 400 frames after initiation. Data displays that SEMORE can find assembly cores, even with high-size heterogeneity including assemblies as small as 94 points up to 7777 points while also correctly dissecting overlapping assemblies. **d,** The corresponding frame of the predicted initiation was used to estimate the offset in time, from growth start to SEMORE prediction, here plotting from 0 to 100 >95% of the distribution for visual clarity. The mean offset of 13 frames, reflects that SEMORE can accurately classify cluster initiation while showcasing the strength of SEMORE in capturing temporal assembly dynamics.

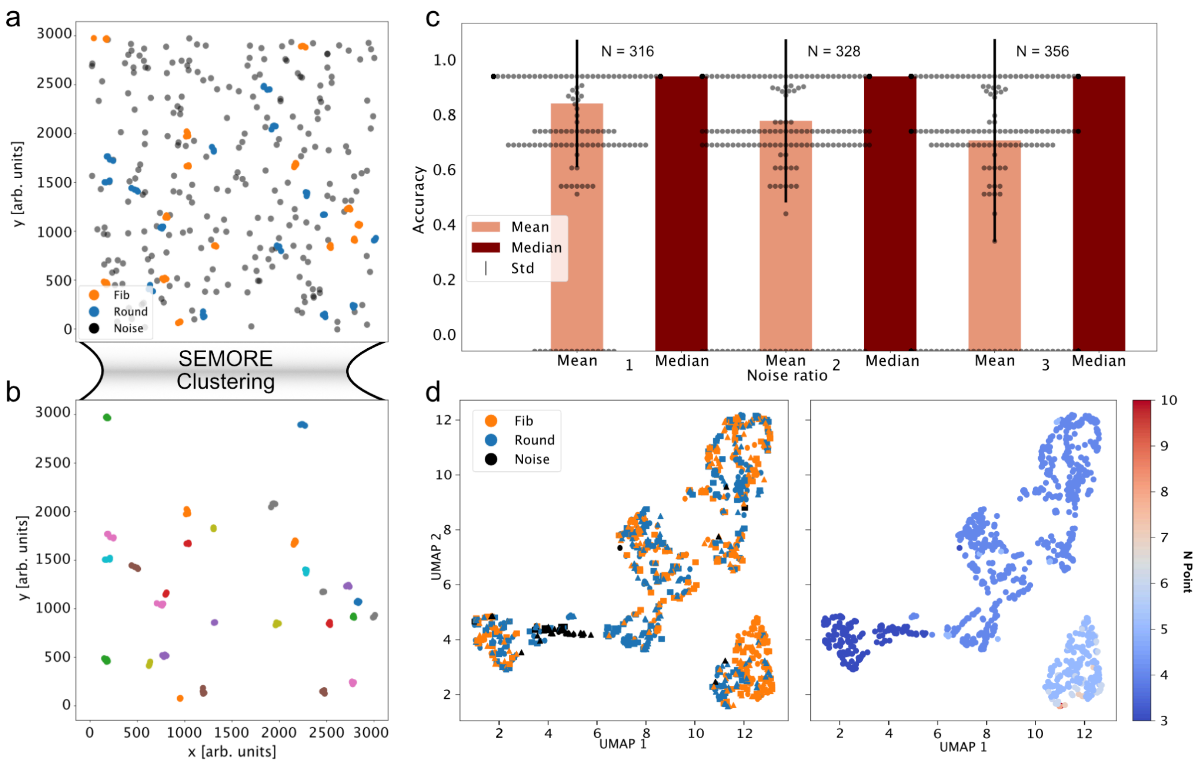

Supplementary Fig. 7: Evaluation of SEMORE' segmentation and structural information extraction on simulated small tetramer assemblies.

a, Simulated experiment containing both fibril (orange) and elliptical (blue) morphology class containing 4 points each. Three noise ratios were simulated 1, 2 and 3 (ratio 2 in figure) with 10 simulations per noise levels each with 30 true assemblies (standard deviations are calculated across each aggregate for the 10 simulations). **b,** Assembly extraction by SEMORE's clustering module. SEMORE's hyperparameters were kept across conditions for comparable results and evaluation of automated analysis. **c,** Accuracy evaluation performed on an aggregate-wise level (see methods) grouped into each noise level. SEMORE achieves accuracies of 90% +/- 23% (noise ratio 1), 84% +/- 30% (noise ratio 2) and 77% +/- 37% (noise ratio 3) showcasing the fidelity of the pipeline even at assembly sizes of four detections with three times as many noisy detections. Throughout all 3 noise ratios the median accuracy is 100% indicating that the found assemblies usually contain all the points of the assembly. **d,** The morphological fingerprints extracted for all structures visualized by an out-of-box 2-component UMAP. Right panel shows wrongly classified structures clustered, notably the noise (black) contained in the lower left corner. Each plotted assembly is represented by its noise to signal level (circle: 1, square: 2, triangle: 3) each noise to signal ratio contains 4, 9 and 38 false positives, respectively, compared to >270 true positives. Presence of false positives is due to true assemblies having densities near identical to noise impeding the smart density filter from accurately excluding the noise density. Left panel shows colour coding each assembly by density revealing true assemblies and false positives can be separated by a single feature from morphological fingerprinting. Proving morphological fingerprinting can aid a non-perfect segmentation, allowing for further correction or focus-based re-segmentation.

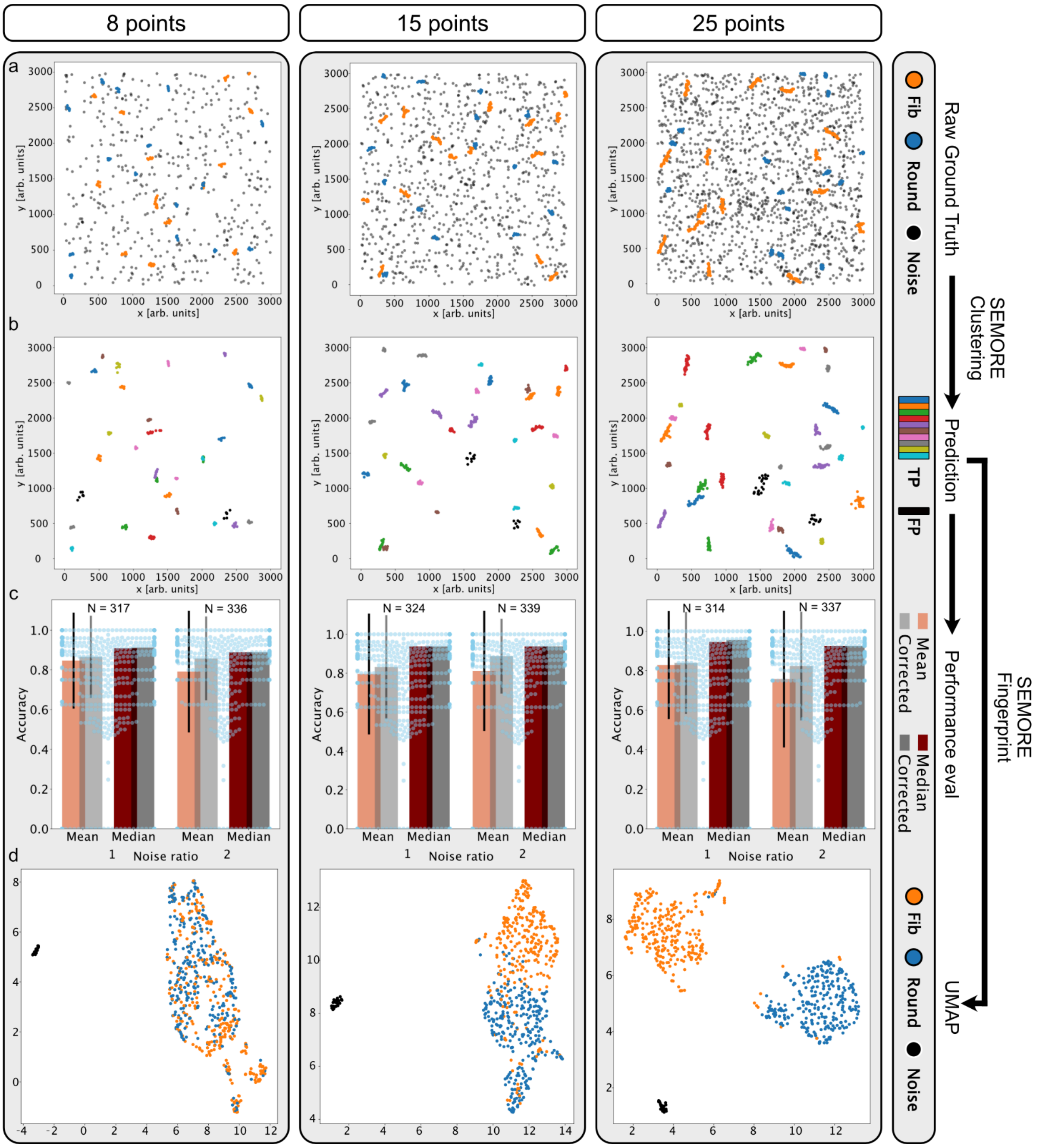

Supplementary Fig. 8: SEMORE classification performance for small, sparse clusters of diverse morphologies.

a, Simulated data containing temporally resolved fibril and static ellipse shape aggregates (coloured orange and blue respectively) containing 8, 15 and 25 points respectively. Each simulated data set contains 30 aggregates equally divided between the two morphology classes, additionally, noise (coloured black) ratios of 1 and 2 (noise ratio 2 depicted in the figure). 10 experiments were simulated for each condition and each noise ratio accumulating to 1800 aggregates over 60 experiments. **b,** Predictions made by SEMORE clustering module, with coloured aggregate being True positives (TP) and black being false positives (FP). SEMORE's clustering module is able to handle the combination of sparse static and temporally resolved assemblies contained within the same experiments. **c,** SEMORE's segmentation performance quantification. Through the SEMORE clustering module an aggregate-wise mean accuracy (see methods) (noise ratio 1 & 2 reported as [1] / [2]) of; 85% / 80% +/- 24% / 31% and a median accuracy of 91%/ 89% for 8 points assemblies, mean 80%/81% +/- 31%/30% with median 94%/94% for 15 point assemblies and a mean 83% / 76% +/- 27%/35% and a median of 95%/93% for 25 point assemblies. As seen in (**d)** the two-component UMAP of morphological fingerprints reveals that all false positive assemblies (noise detections) are perfectly separated. Using this separation as correction accuracies improve: mean 87% / 86% +/- 21% /21% median 91% / 89% for 8 points, 83% / 89 % +/- 27%/20% median 94% / 94% for

15 points and mean 84% / 82% +/- 26% / 27% median 96% / 93% for 25 points. Performance is improved proportional to noise-ratio as the higher noise levels induce more false positives which in turn are corrected by morphological fingerprinting. **d,** Two-component UMAP embedding of the corresponding fingerprints with the different noise-ratio combined within point types (N = 611, 611, 586 for 8, 15, 25 points respectively).

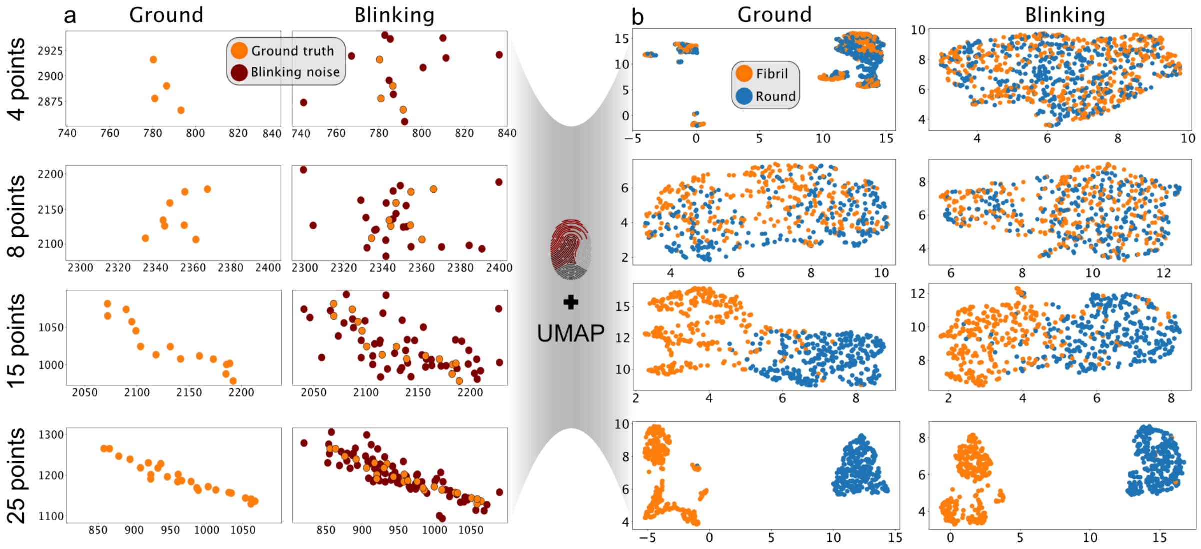

Supplementary Fig. 9: Effect of blinking on SEMORE's morphological fingerprinting and characterization of morphological classes.

Each row in **(a)** and **(b)** are grouped and depict a certain simulated structure size (4, 8, 15 and 25 data points, respectively), all axis dimensions are in arb.u . **a,** Left side shows detections from representative fibril structures as "Ground truth" (orange) prior to blinking and the right side depicts the corresponding structures obtained after blinking (dark red). These post-blinking structures do not include the ground truth points (orange). For each row, 600 ground truth structures were simulated along their corresponding blinking counterpart and as performed in SI fig. X two types of morphologies were simulated in equal amounts, namely fibril-like and spherical. **b,** The morphological fingerprints of each structure are visualized by a 2-component UMAP with colour-coded by morphology type. The ground truth structures (no blinking) shown on the left side of **(b)** and post-blinking structures on the right side. The results show distortion in separability induced by blinking is positively correlated with the number of detections with a given structure as expected as blinking will dominate the morphology of small structures. From 15 detections in a structure and above blinking has little to no effect on the morphological fingerprinting module of SEMORE.

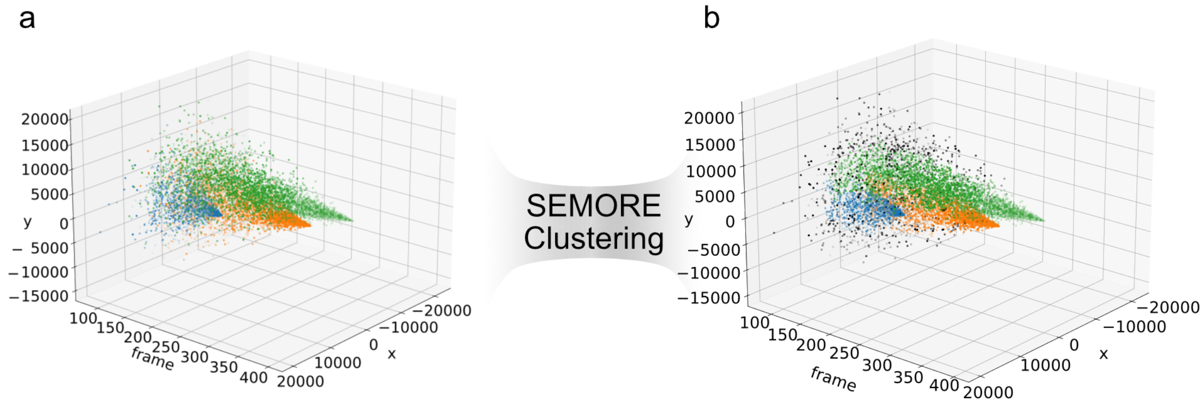

Supplementary Fig. 10: Demonstration SEMORE clustering of morphology shrinkage.
a, Demonstration of 3 simulated isotropic structures that while proving a challenge due to spatial overlapping also undergo shrinking (colour-coded by identity). **b,** Results show SEMORE accurately identifies spatially overlapping clusters reducing in size at all frames in the simulation allowing the tracking of individual shrinkage in time. As shrinkage is the direct opposite of growth, SEMORE's clustering module is directly applicable as is after a simple preliminary step of flipping the temporal axis of the simulation or experiment.

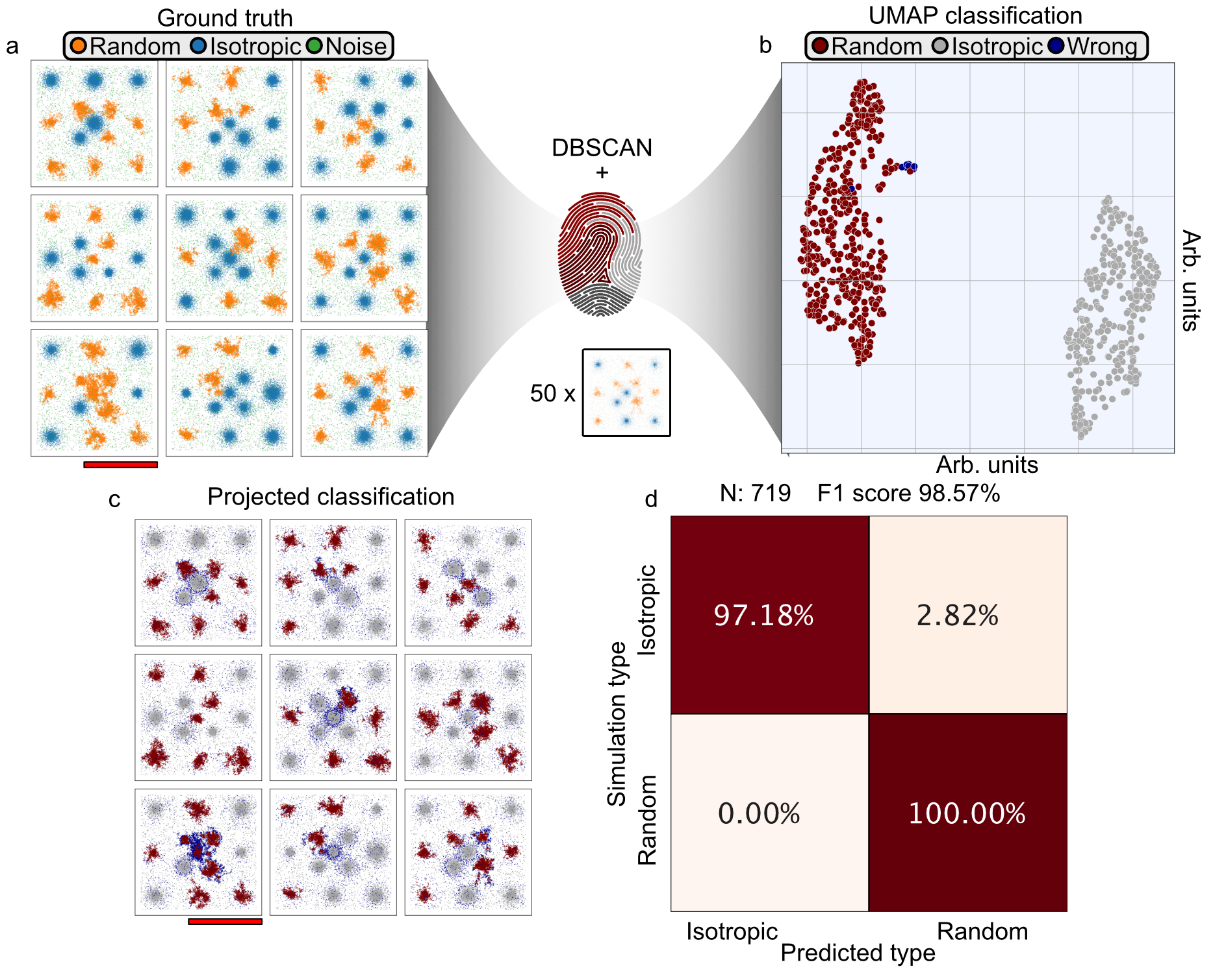

Supplementary Fig. 11: Classification capabilities of unsupervised morphology fingerprinting, in high-density regions of diverse aggregate morphologies for SMLM without temporal features.

a, 9 representative simulated experiments of the total 50, each containing 13 aggregates with the same start seed location throughout the simulations. The aggregate type is randomly selected as either isotropic or random and colored accordingly. Scalebar in red 40000 arb.u. **b,** To mimic an analysis pipeline of SMLM data without temporal resolution, each simulation is treated through a DBSCAN (eps = 500, min_sample = 25) to segment the contained aggregates. The SEMORE fingerprint module is then used to extract the morphology fingerprint for each of the extracted structures. The resulting features are then embedded through a UMAP (n_neighbors = 15, min_dist = 0.1) and clustered in the embedded space through another DBSCAN (eps = 1, min_sample = 10). This results in 2 clearly separated clusters used to predict the dominant aggregate type contained within, with Random colored dark red, Isotropic colored gray and wrong prediction colored blue. **c,** The corresponding prediction colored on the raw data, for visual comparison while still coloring incorrect prediction as blue. Scale bar in red is 40000 arb.u. **d,** Confusion matrix for the binary prediction of aggregation type, achieving an F1 score of 98.57% indicating almost perfect predictions and information capture within the fingerprint.

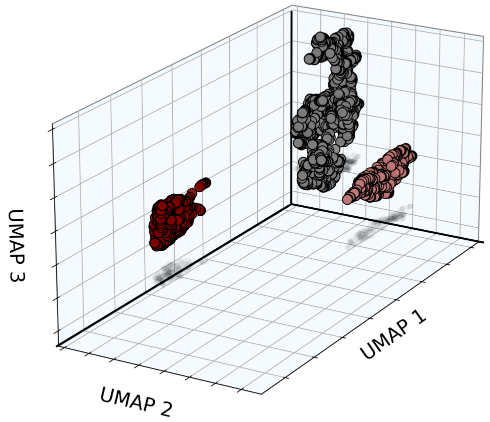

Supplementary Fig. 12: Resulting UMAP from SEMORE clustering applied on the simulated structures with the smart density filtering.

This results in 3 separate clusters colored according to the color mapping as seen in Fig. 3b. However, as the smart density filtering removes the “noise”-aggregate the noise cluster is not present in the UMAP, which is a testimony to the utility of the smart density filter.

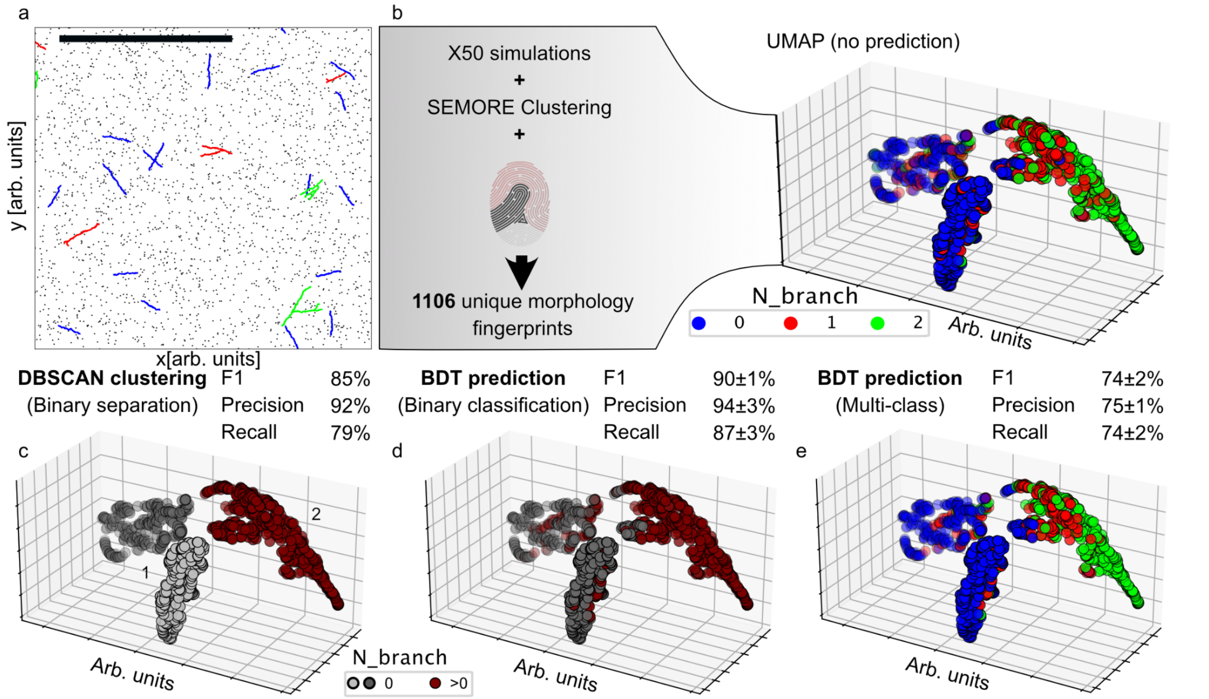

Supplementary Fig. 13: Fibril branch classification performance through feature-class specific investigation.

a, Representative examples of the simulated experiments with the fibrils colored according to their number of branches. Black scale bar 40000 arb.u. **b,** Treating the 50 simulated experiments results in 1106 unique fibril morphology fingerprints. These were embedded through UMAP (n_neighbors = 15, min_dist = 0.1, n_components = 3) and each point colored to its corresponding aggregate branching degree. **c,** A DBSCAN (eps = 1, min_sample = 10) clustering performed in the embedded space for binary classification of non-branching (positive) and branching (negative) fibrils. The results yielded three distinct clusters: Gray clusters being highly dominated by non-branching fibrils and collectively representing cluster 1, whereas cluster 2 in red is highly dominated by branching. This binary classification results in an overall F1 score of 85%, with a recall of 79% and a precision of 92 %. **d,** To demonstrate the versatility of the morphology fingerprinting, a boosted decision tree (BDT) was fitted and evaluated through a k = 5-fold cross validation, achieving a mean F1 score of 90±1% which increased to 94±2% when exposing all features of the morphology fingerprint. **e,** Furthermore, the same BDT model was fitted to classify the multi-class problem of N_branches, resulting in a mean F1 score of 74±2% increasing to 82±3% with the use of all features. The overlap of classes seen in the embedded space can be caused by multiple factors, as the investigated feature class only contains information regarding morphology. This results in the blinding of fibril outliers, e.g. late branching as seen in red aggregates in (**a)** or highly diverse angle distribution in back-bone growth. This would also explain the low but prominent increase in BDT performance through all the features compared to only circularity which comprehensively contains 5 out of 40+ features.

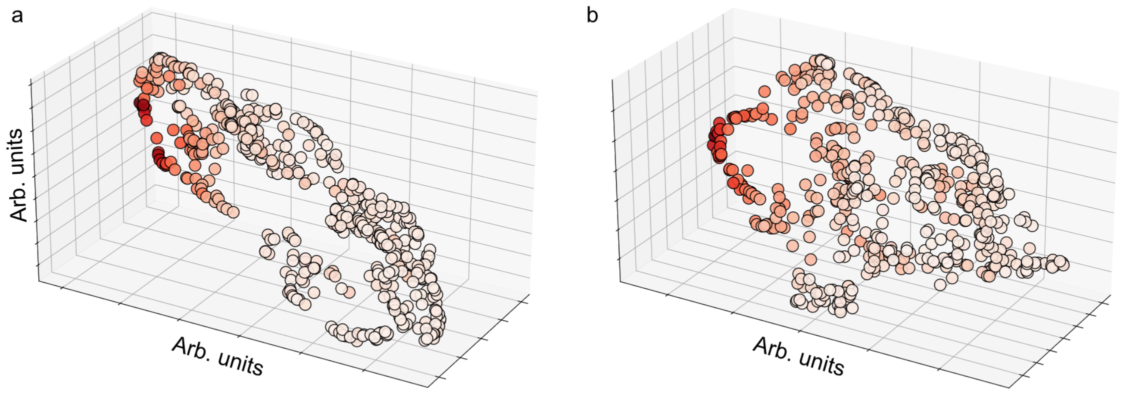

Supplementary Fig. 14: Simulation type-specific UMAP investigation based on the circularity feature class.

a, Corresponding embedded features from simulated isotropic aggregates, the general structure of the embedding depicts a linear dependency on the aggregate morphology due to no fundamental changes in growth pathways, as compared to the branching behavior in fibrils. The comparably narrow spread in points is due to the segmentations not being perfectly extractions and the high diversity in size. **b,** The embedding for simulations based on steric hindrance ("random" aggregate) has a wider spread out due to stochasticity in their s growth kinetics and morphology. Both the UMAP and features used are the same as seen in Supplementary Fig. 5, with the datapoints color corresponding to the variance-feature value, with darker color being a higher value.

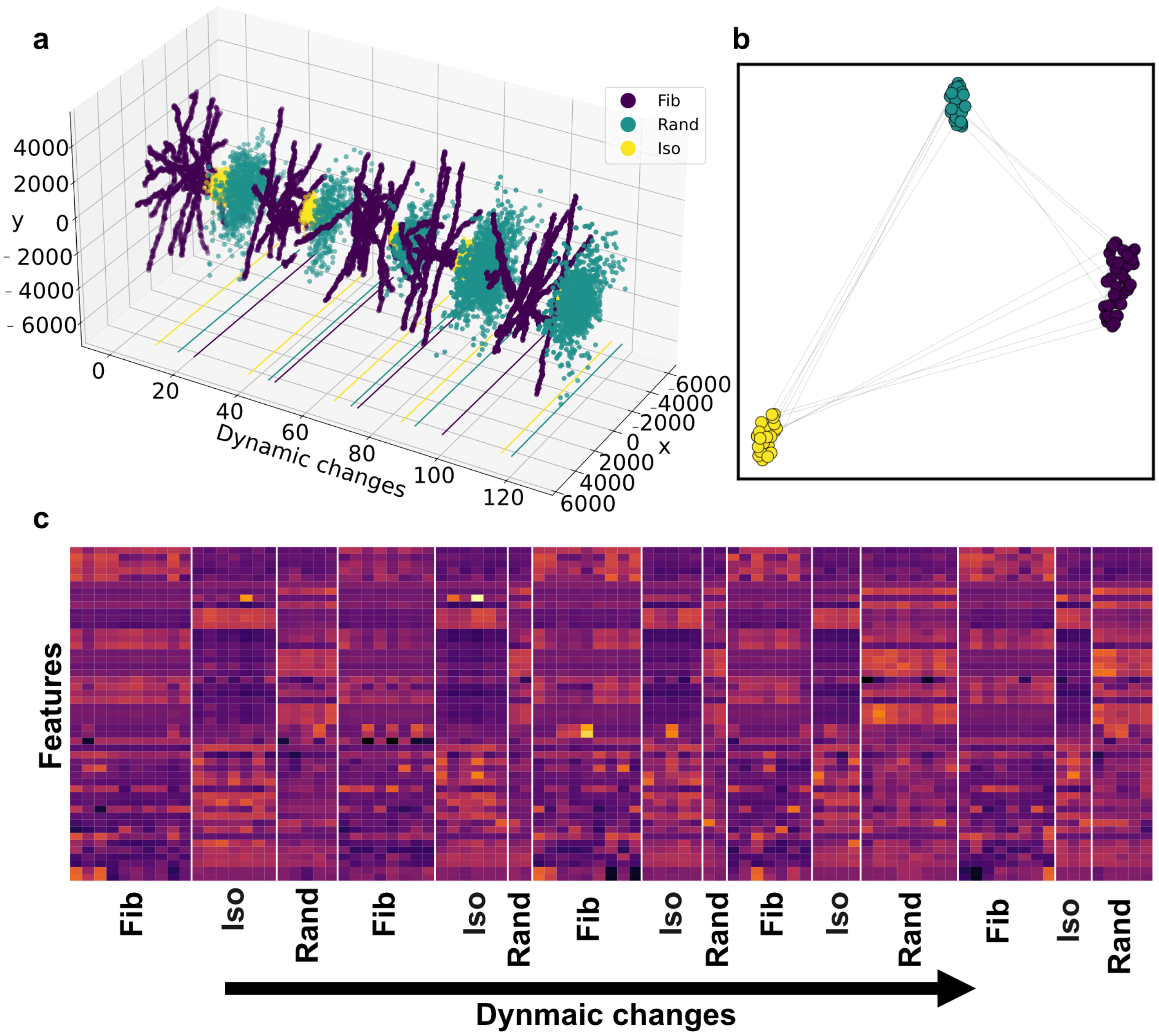

Supplementary Fig. 15: Depiction of recurrent SEMORE fingerprinting for dynamic morphology variation of protein clusters.

a, Simulated dynamic morphology variation of protein assembly by sequentially exhibiting structures with diverse morphologies. All temporal states consist of 200 points colour-coded by morphology type. Lines projected at the bottom represent a new morphology initiation (coloured by morphology type). For each temporal state, the SEMORE morphological fingerprinting module was used to extract features for probing the morphology changes across temporal states. **b,** The morphological fingerprints were embedded by an out-of-the-box 2-component UMAP resulting in a clear separation of the morphology class across time (with time linked by grey lines). Systems with more or less gradual change of morphology would result in a more or less regressive separation. **c,** MinMax transformed fingerprints plotted into a heatmap with each column representing a temporal state and white lines indicating drastic morphology type changes. Each row represents a morphological feature, in the same order as seen in Supplementary Table 1. The heatmap rows showcase the morphological fingerprinting module's ability to capture the mechanistic of temporal states and map the overall features changes involved in major changes in morphology.

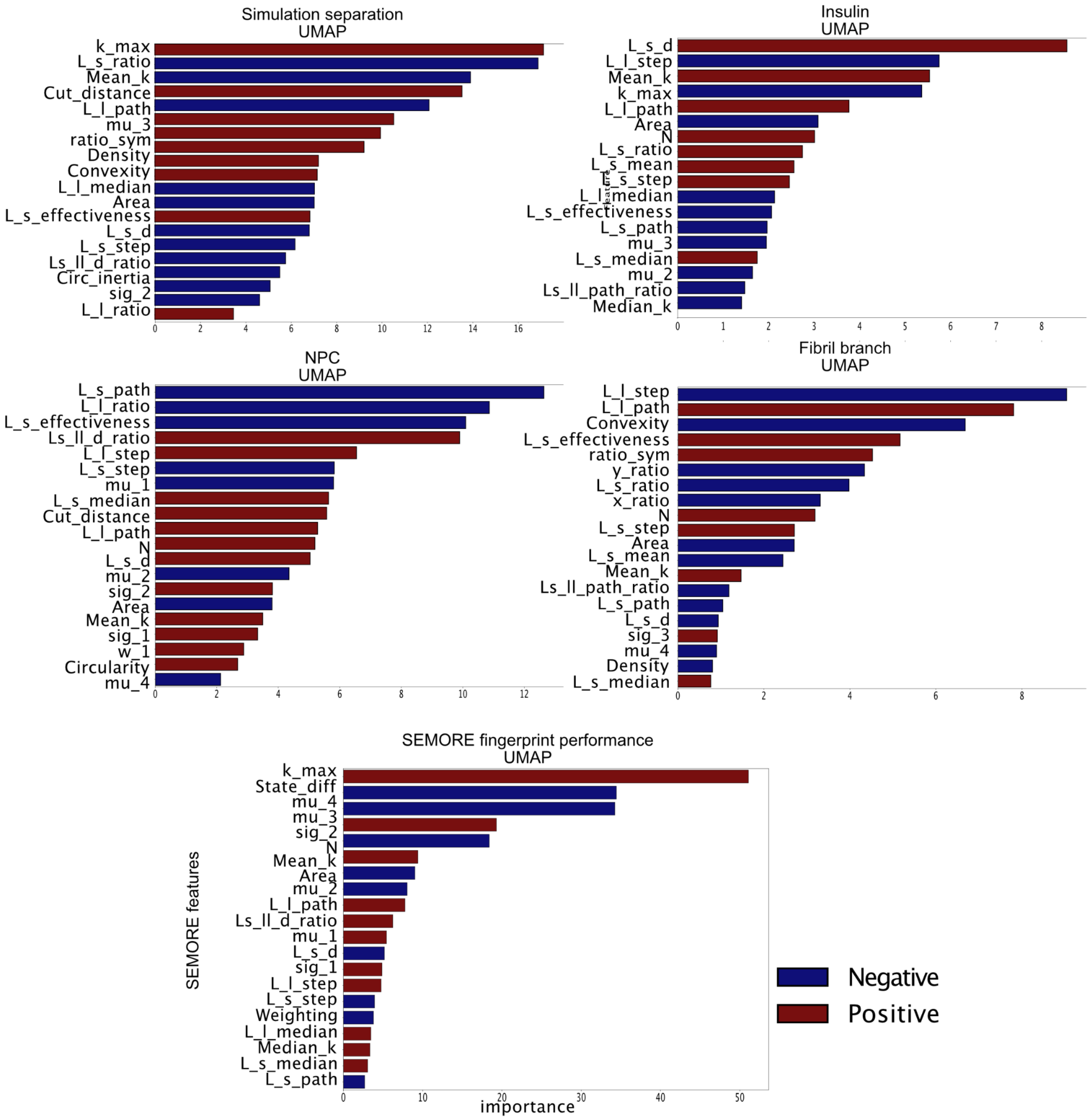

Supplementary Fig. 16: UMAP feature importances.
Extracted feature importance of all UMAPs presented in the article, the bars are colored according to the weighting (positive: red, negative: blue) and the importance values are found through linear discriminant analysis (LDA) fitted to the predicted labels.

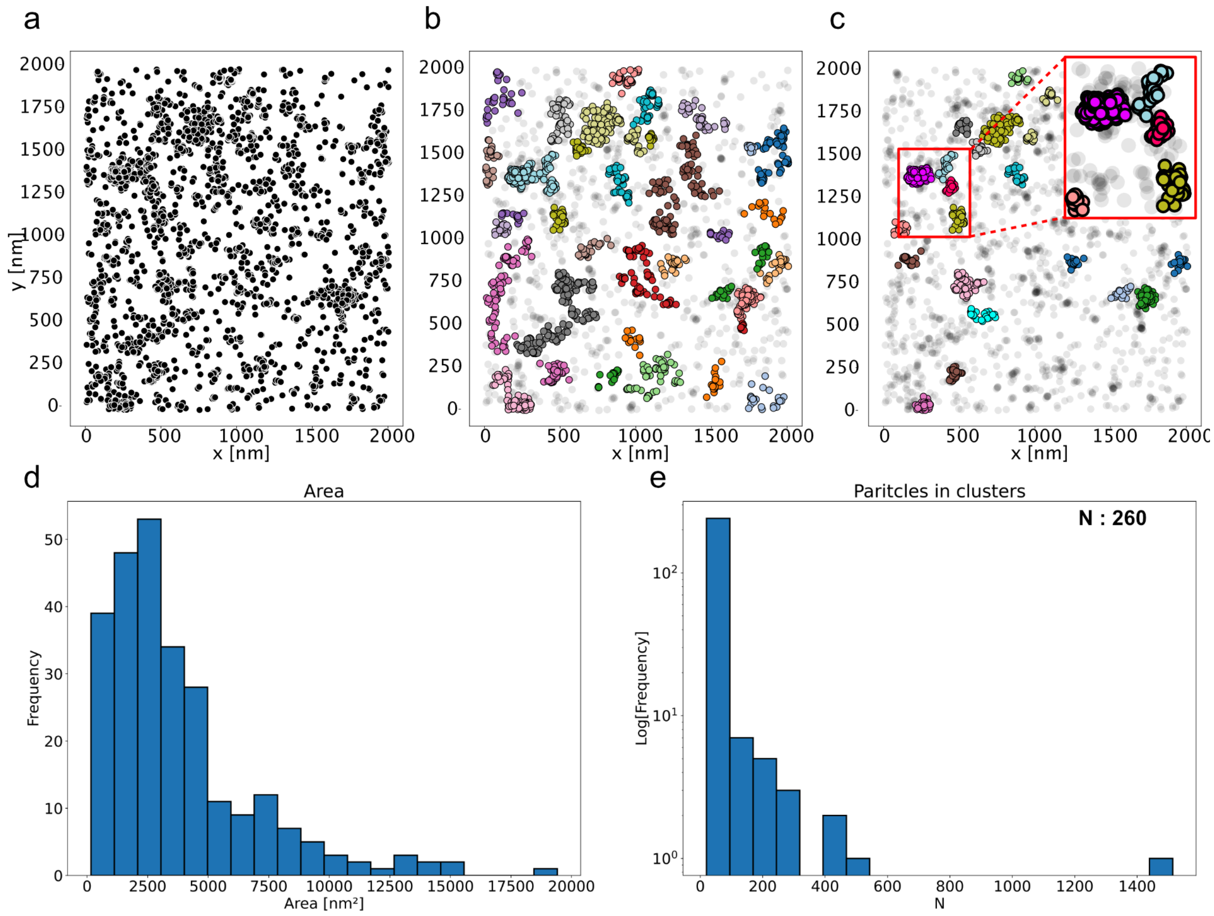

Supplementary Fig. 17 dSTORM data from Nieves et. al[1] clustered and quantified by SEMORE.
a, Raw detections from SMLM data of fibroblast growth receptor 1 (FGFR1) on a MCF7 cell presented by Nieves et al. **b,** Initial clustering by SEMORE's clustering module using the inherent data-driven model choice of HDBSCAN (Min_cluster_size = 15 and Min_sample = 5). Each localization is colored by its SEMORE annotation, with black representing noise and all other colors representing captured clusters. **c,** The final clustering by SEMORE after refinement and smart density filtering (see Methods). Localizations are colored corresponding to the final SEMORE prediction. Red box depicts the same zoom-in as seen in Nieves et al inside which they report 2 unique clustering indices similar to the initial clustering seen in **(b)**. SEMORE's additional refinement split one of these into four distinct clustered indexes, thus resulting in five clusters. These settings are constant through the 15 datasets from Nieves et al. **d,** Histogram of cluster area estimation. SEMORE identifies mean cluster area of 0.004 ± 0.003 μm^2 and median of 0.003 μm^2. SEMORE provides more fine-grained area calculation than convex hull (see Supplementary Fig. 20) and may split larger clusters to smaller during rounds of refinement. **e,** Distribution of points contained in the proposed clusters with a mean of 52 ± 108 and a median of 29. SEMORE and Nieves et al. obtain qualitative similar results and have 88% agreement in assigning points as noise showing the general agreement as methods.

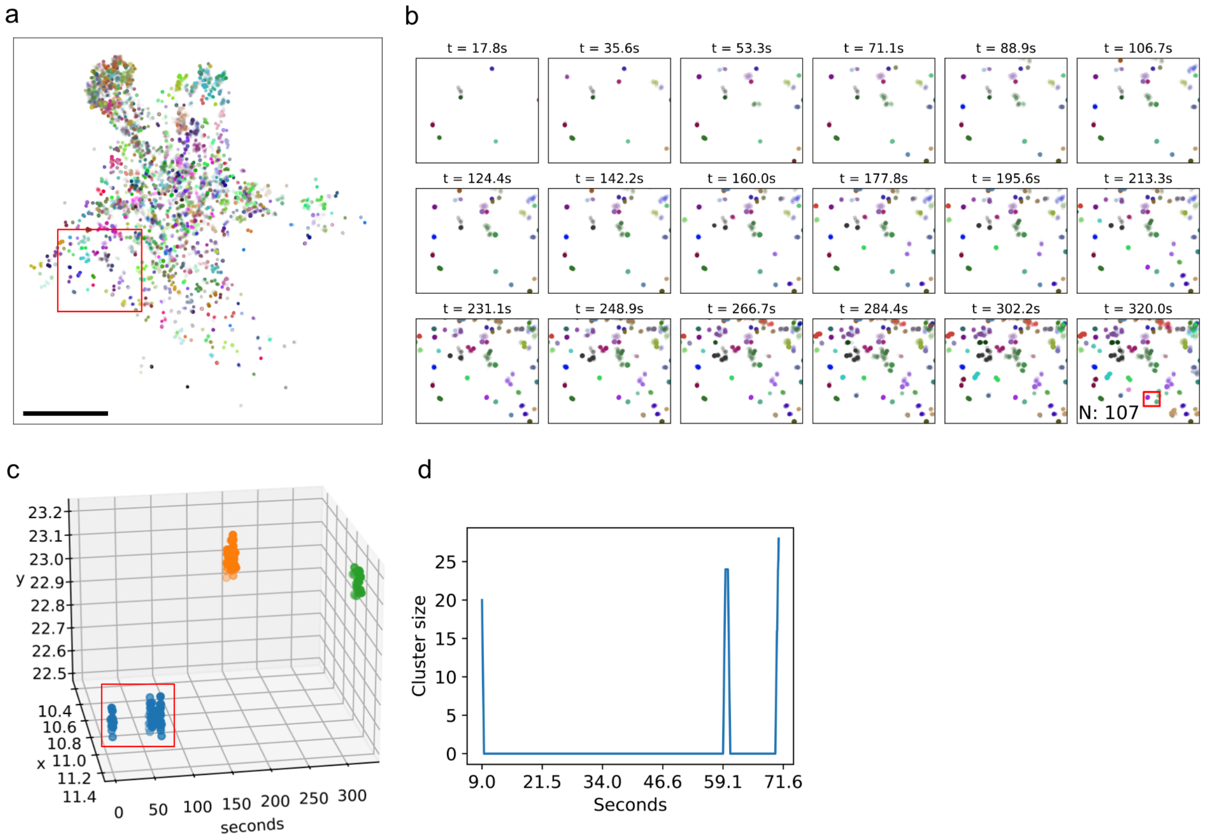

Supplementary Fig. 18: Evaluation of SEMORE on temporarily resolved Sx1a-mEos2 sptPALM data[2].
a, Raw localizations of all detections from tracks longer equal or above 20 frames of Sx1a-mEos2 colored in grey for noise detections and other colors for individual clustered detections captured by SEMORE (HDBSCAN; min_cluster_size: 10, min_samples: 10, cluster_selection_epsilon: 0.04) for the entire field of view (sptPALM traces above length 20) showing near identical results to Wallis et al[2]. Red box indicates a region of interest. **b,** Snapshots in time plotting all detections prior to the indicated acquisition time from the region of interest in **(a)** showing SEMORE captures the time-resolved appearance and growth of clusters. Last snapshot shows N=107 total identified clusters by SEMORE as compared to 104 manually counted clusters in the same snapshot seen in Hou et al. The snapshot contains a red box highlighting an additional region of interest. **c,** 3D (xyt) plot of the region of interest presented in **(b)** showing the temporal morphological changes of clustered detections. Red box indicates a hotspot region in space with repeated clustering of detections in time. **d,** Shows the temporal evolution of cluster size for the region of interest in **(c)** showing SEMORE's successful capture of the repeated clustering of detections also discussed in Wallis et al[2].

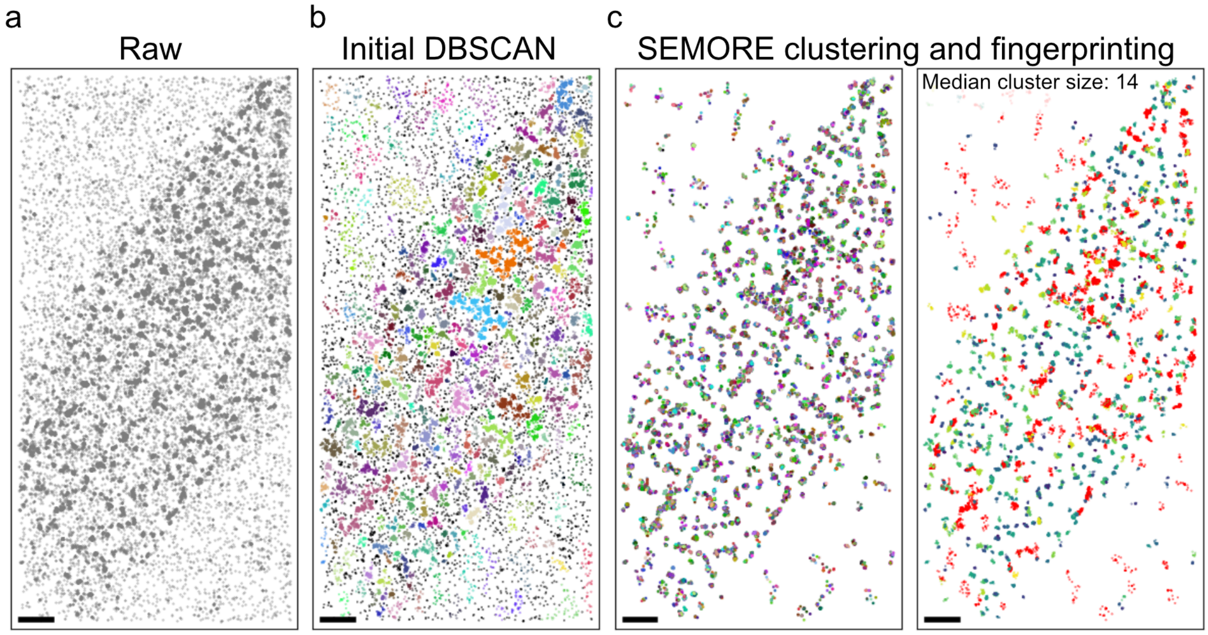

Supplementary Fig. 19: Evaluation of SEMORE on temporarily resolved live-cell PALM data of ryanodine receptors (RyRs)[3].

a, Raw localizations of all detections of RyRs from live-cell PALM from Hou et al[3] colored in grey. Scale bar 2 microns. **b,** Initial clustering of data by DBSCAN showing clusters colored by cluster identity. **c,** left panel: Final clustering by the full clustering module of SEMORE utilizing the smart density filtering and temporal refinement with individual clusters colored by identity (DBSCAN; dbsmin_samples: 10, SEMORE: final_min_points: 10, rough_min_points: 10, investigate_min_sample: 10). Hyperparameters of SEMORE chosen to capture the relatively small clusters formed by RyRs. Results show how SEMORE obtains granularity otherwise infeasible by DBSCAN alone. Right panel: Final clusters from SEMORE colored by the “longest shortest distance” feature from the morphological fingerprinting module with clusters exceeding a threshold of 400nm colored in red showing how SEMORE can capture RyR cluster morphology and separate noisy detections. Utilizing the tight packing assumption for RyRs[3] the RyR clusters identified by SEMORE reports on average 18 RyR with a median of 14 RyR in line with the 9 RyR reported by Hou et al importantly achieving so without any intensity thresholding.

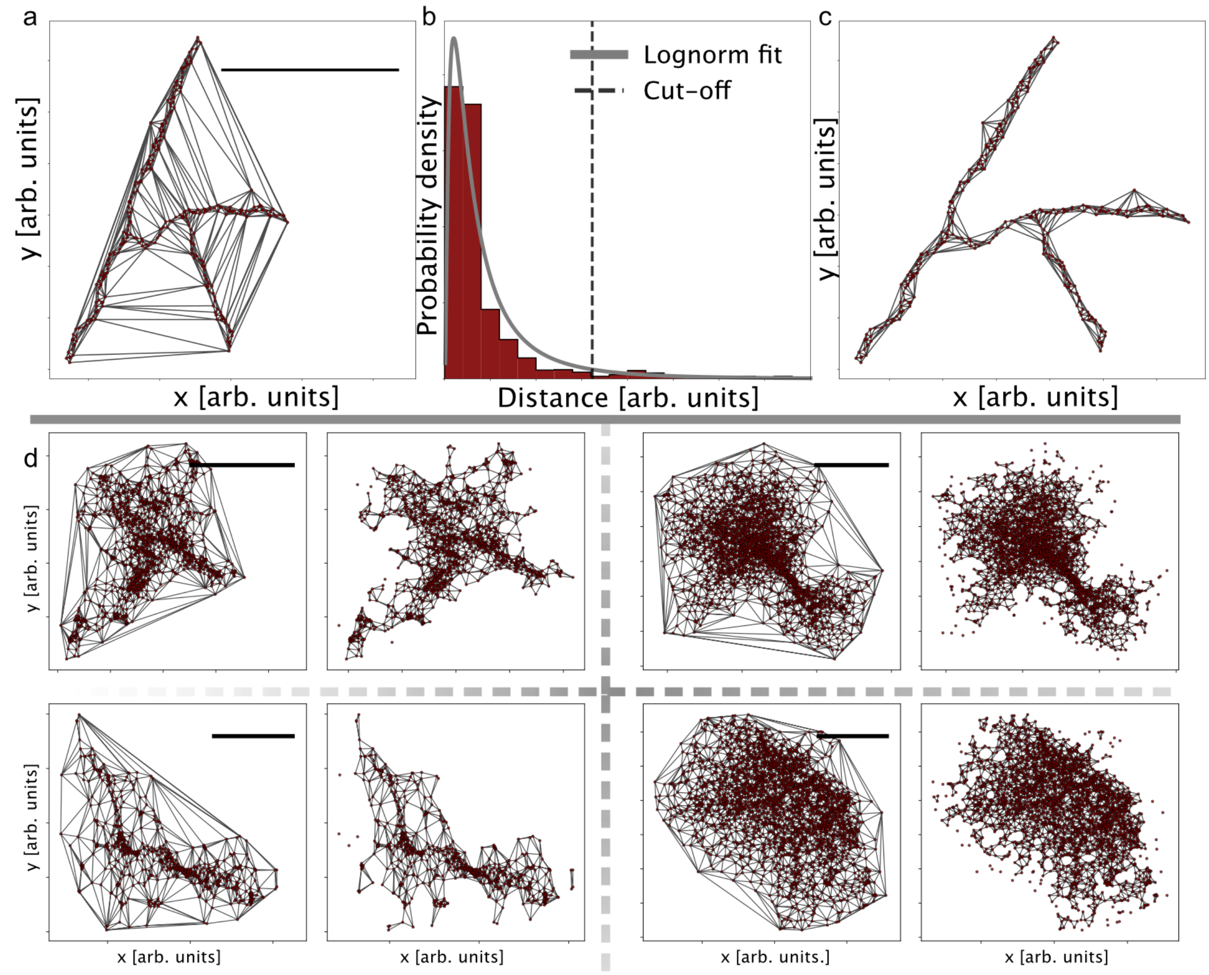

Supplementary Fig. 20: Demonstration and visualization of structure-polygon for size estimation.
a, Displaying a simulated fibril structure, along the collected localizations (dark red) and Delaunay triangulation (grey). **b,** The triangle-edge distance distribution is fitted by the lognormal. From this, a distance threshold is calculated as the right-tail 5% probability or below. **c,** The threshold is then used to prune the triangle edges, from which only the remaining closed triangles are used to calculate the area of the given structure. **d,** Shows the Delaunay triangulation along its pruned counterpart from which the area can be evaluated, both as a static image or temporally as the structure grows. Scale Bar 5000 arb.u. for **(a)** and 5000 nm for **(d)**.

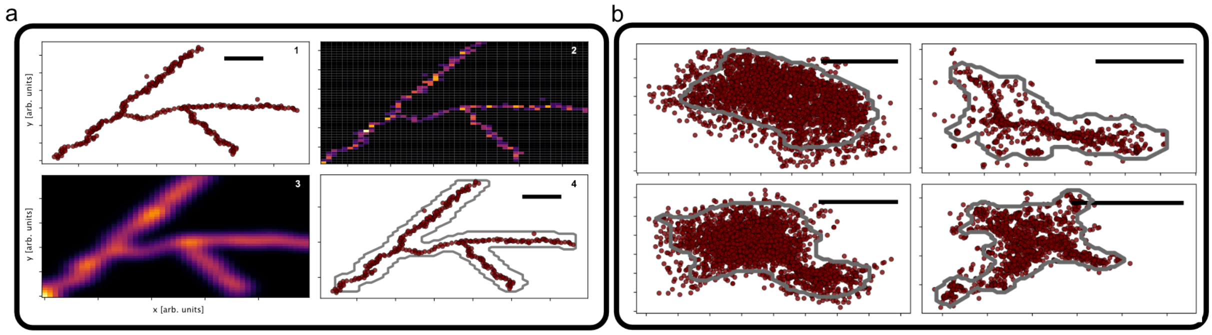

Supplementary Fig. 21: Pipeline for morphology edge estimation.

a, The 4-step process for achieving the circumference estimate of a given structure a. 1) The x, y coordinates of the investigated structure are collected, 2) the data points are fed into a 2-dimensional 50x50 bin histogram, 3) a Gaussian blur kernel convolved over the counts in the histogram to create a smoother structure. 4) From a pre-defined percentile threshold (70% default), a binary mask is acquired and extracted through a one-level contour analysis. This contour can then be used along with the pre-defined core to calculate the corresponding features of the circularity sub-set. For fine-tuning, the number of bins, sigma of the Gaussian kernel and the percentile cut-off are adaptable, while for a density-dependent circumference, the amount of connection acquired from the graph network can be fed into the initial histogram as weights (a.2). **b,** For clarification the method is visualized for the same 4 random insulin aggregates as in Supplementary Fig. 9. Black scale bar 1000 arb.u. for **(a)** and 1000 nm for **(b)**.

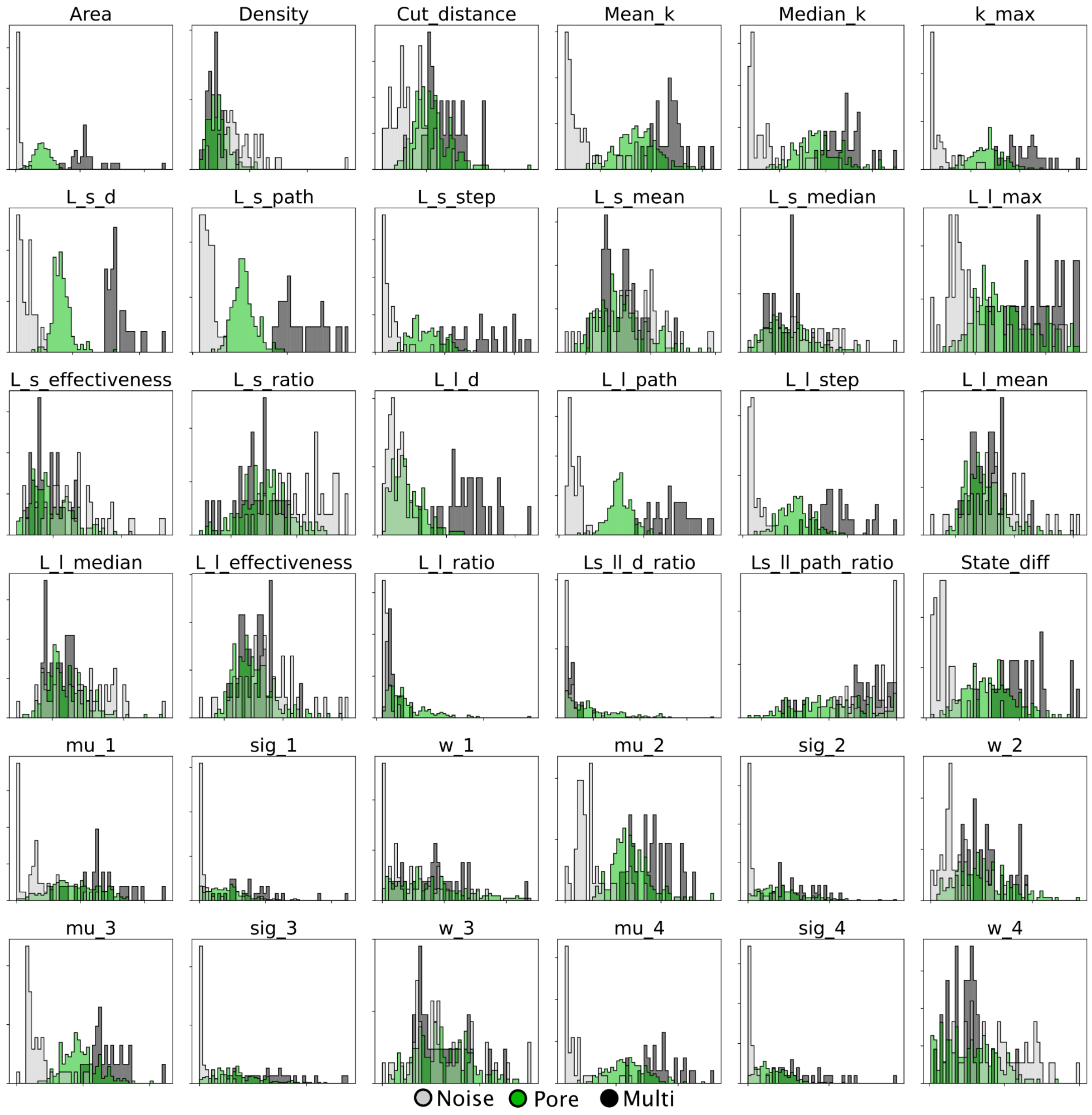

Supplementary Fig. 22: All the used spatial features distributions from nuclear pore complex treatment from the NPC-A647 dataset[4].

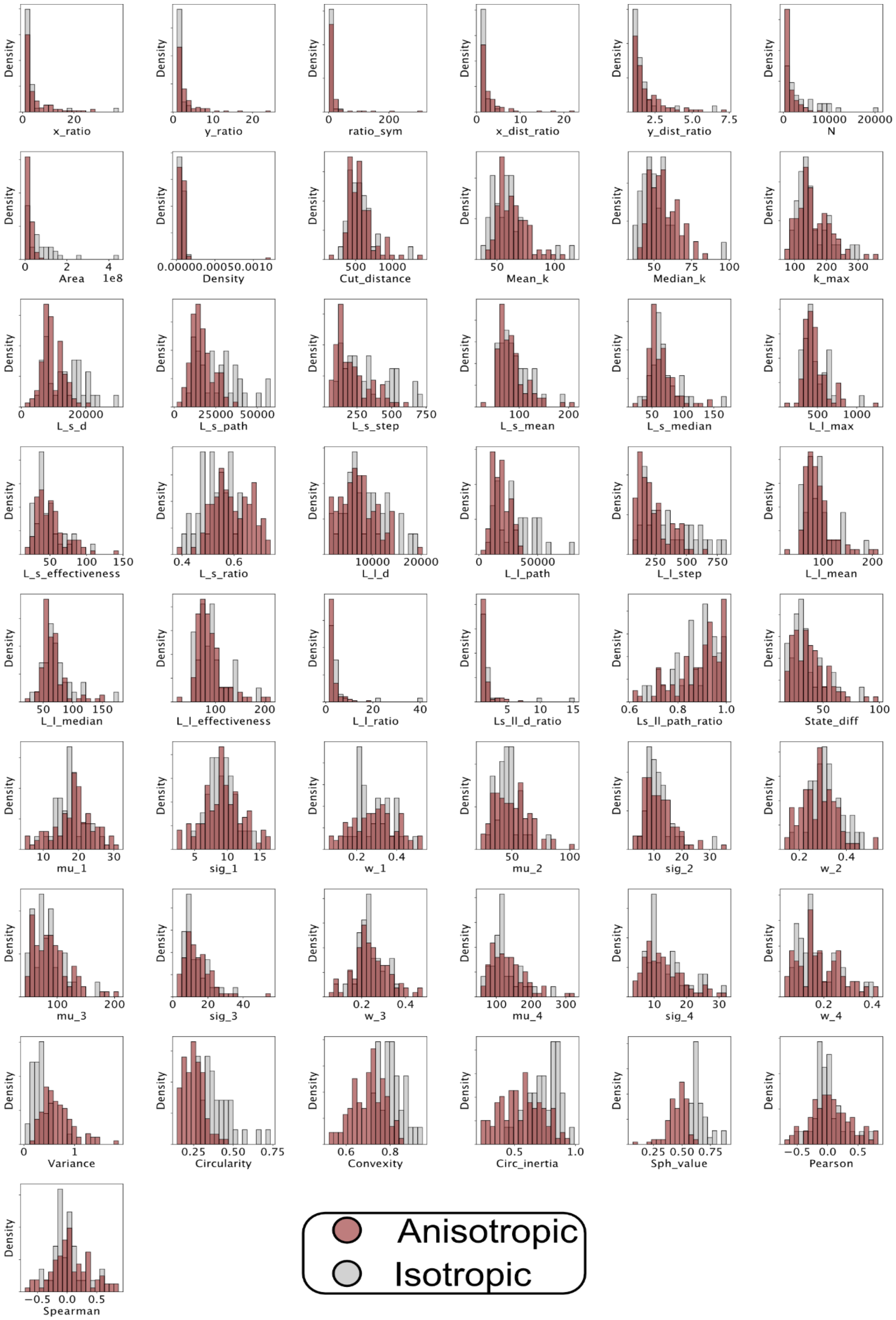

Supplementary Fig. 23: Morphology fingerprint feature distributions for anisotropic and isotropic growth type aggregates from the insulin aggregation studies by REPLOM[5].

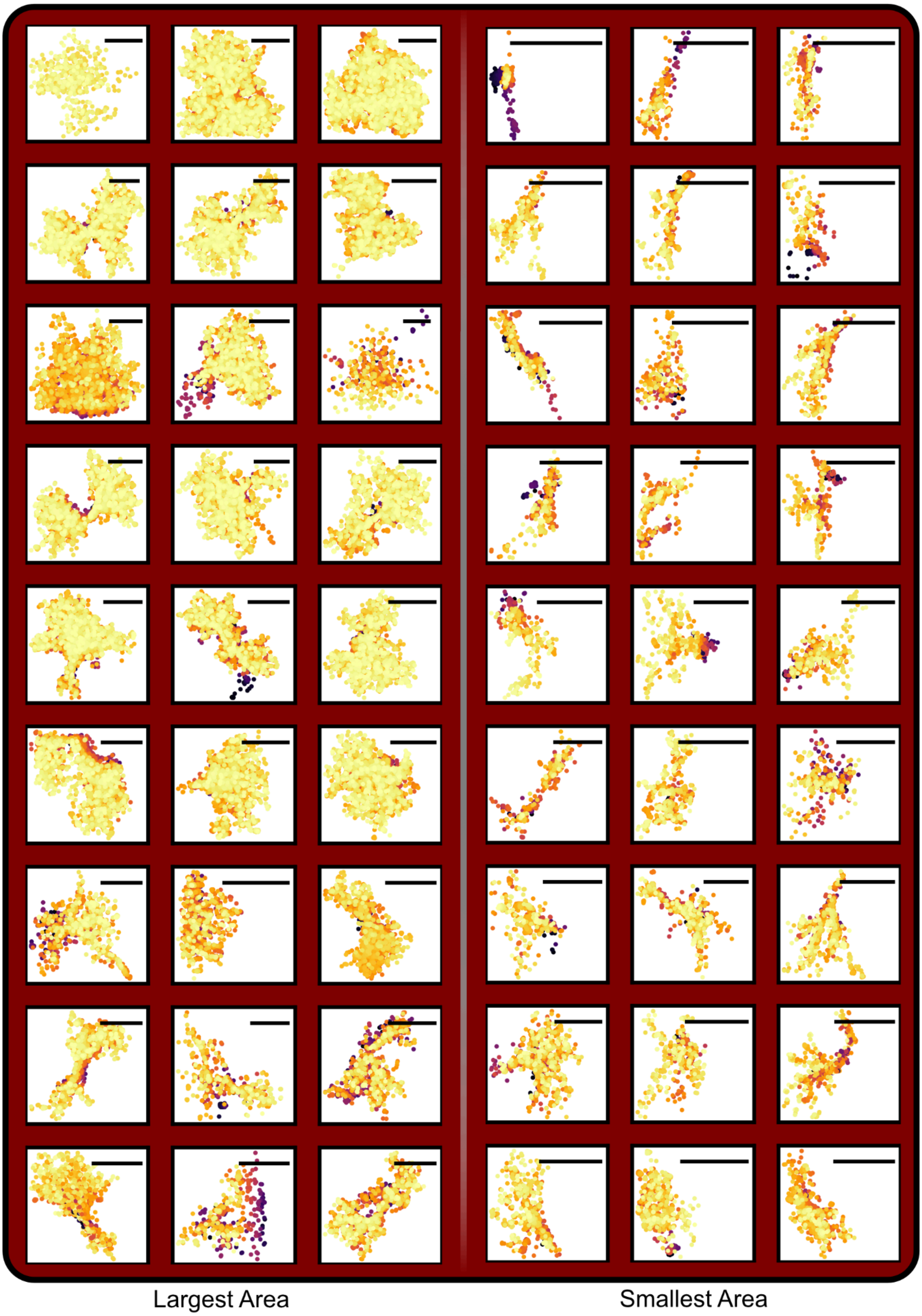

Supplementary Fig. 24: The 27 aggregates with the biggest and smallest areas of the anisotropic classified structures from the insulin aggregation studies by REPLOM[5].
Black scale bar is 5000 nm.

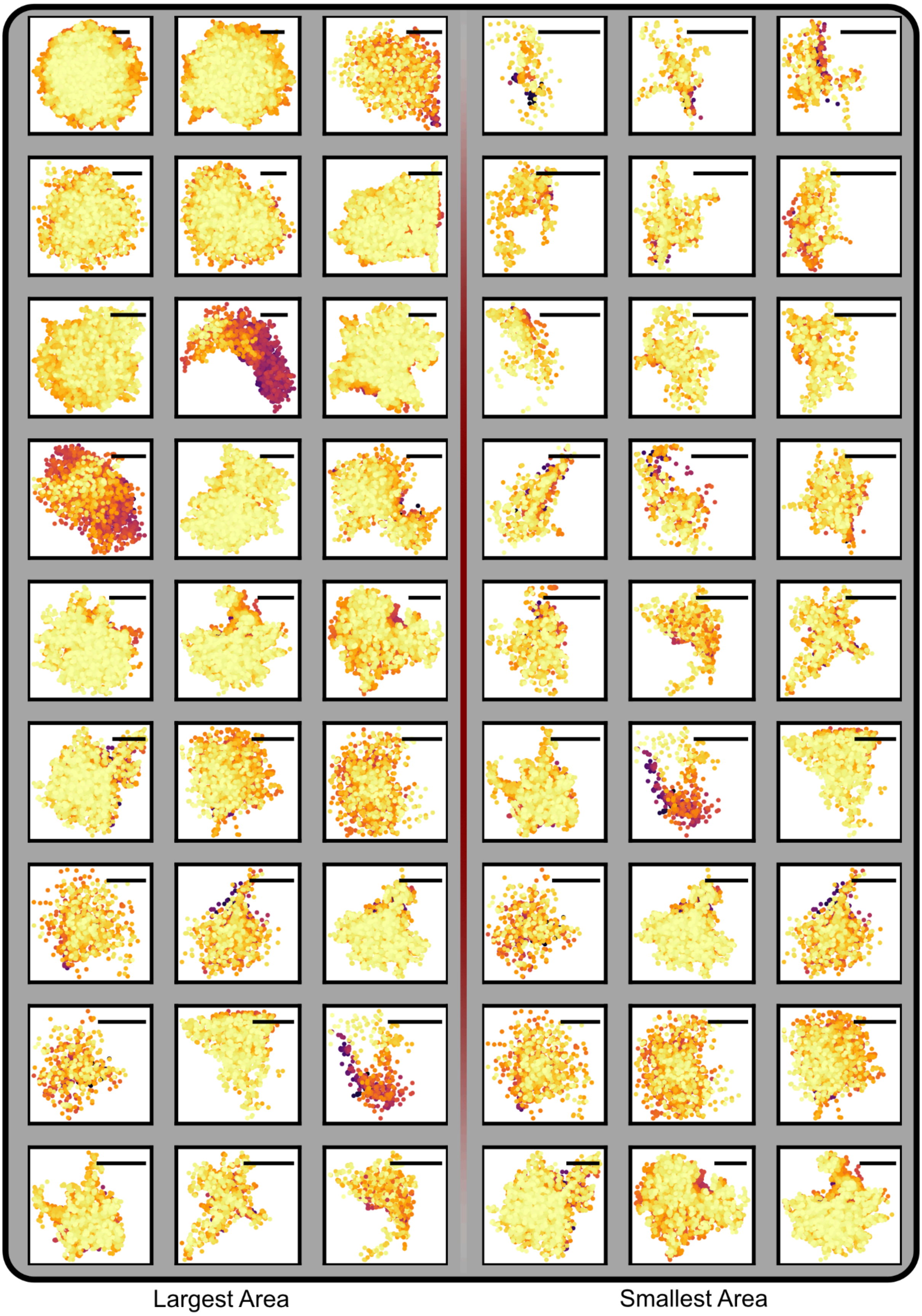

Supplementary Fig. 25: The 27 aggregates with the biggest and smallest areas of the isotropic classified structures from the insulin aggregation studies by REPLOM[5].
Black scale bar is 5000 nm.

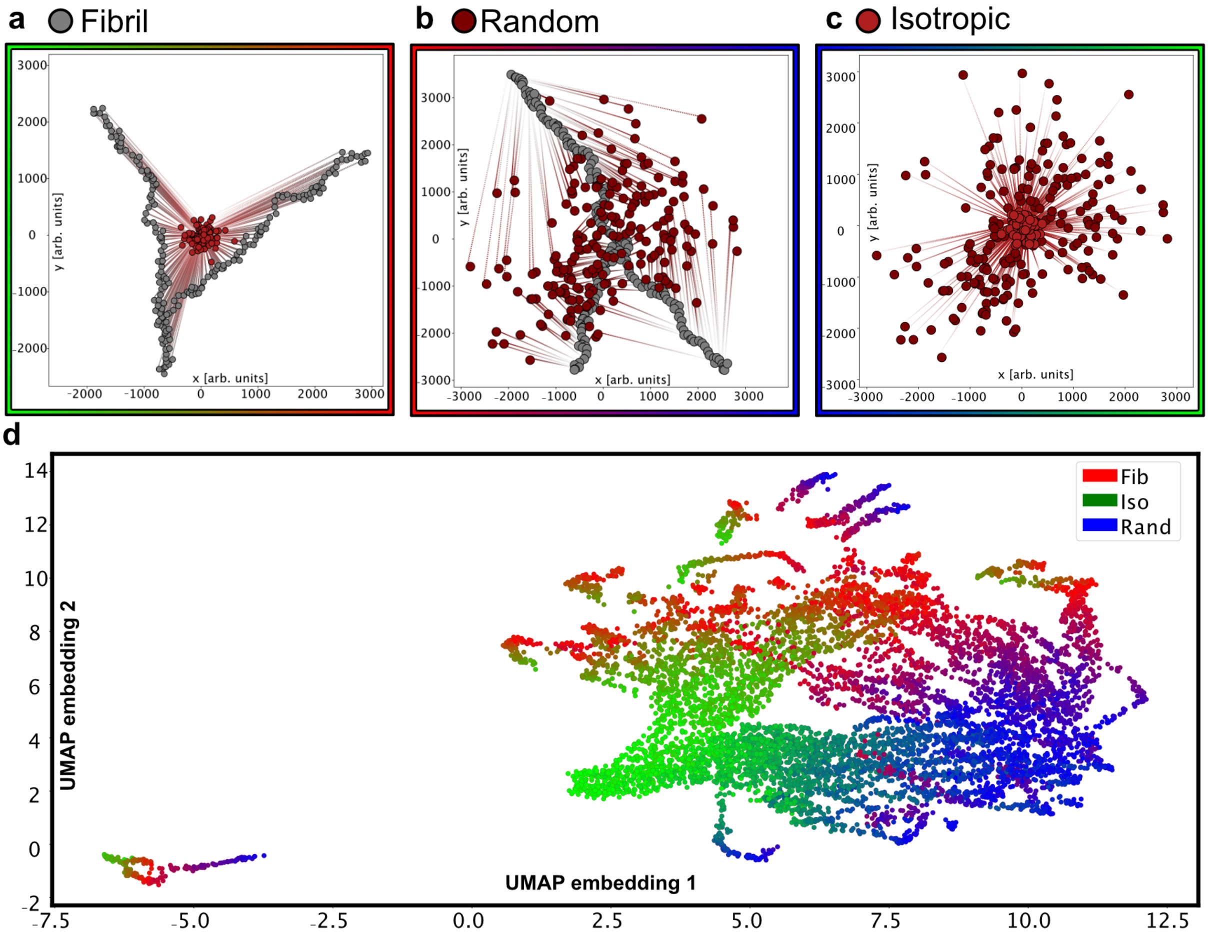

Supplementary Fig. 26: SEMORE's Morphological Fingerprint captures gradual transitions in morphology.

Thirty aggregates of each morphology class (fibril-like: fib, isotropic: iso, sterically-hindered/random: rand) of equal size were simulated resulting in ninety distinct structures which are placed sequentially in random order whilst ensuring no consecutive types. Between each of the ninety structures (89 transitions) 100 positions are constructed from interpolation (see methods). Thus, a total of 8900 intermediates gradually changing morphology between the three structures were evaluated using the fingerprinting module of SEMORE. All resulting transitions are connected in time, meaning the final structure of the (i) transition is the starting structure of (i+1) transition, thus creating a single dynamic structure of gradual transitions. **(a-c)** Shows representative transitions from fibril to isotropic, from fibril to random, and from random to isotropic, respectively. The lines drawn between points in **(a-c)** represent the 100 different positions expressed throughout the interpolation from the initial structure to the target structure. There are two colour gradients on each panel in **(a-c)** both representing the percentage of morphology transition between the three distinct morphology classes. Which is also used in **(d)**. **d,** To visualize the general data manifold of the morphological fingerprints across the 8900 structures, the high dimensional morphological feature set was dimensionality reduced through a UMAP (n_componnents : 2, n_neighbors: 400, min_distance: 0.5). Similar to Supplementary Fig. 15 the embedding shows capture of distinct morphology classes and now also the continuous gradual change between the distinct morphology classes, as each structure morphs into the next. Thus, highlighting SEMORE's strength in capturing gradual morphological evolution. 97.9% of data are grouped in the continuous flow between the three distinct morphologies (x=7.5, y=6). The small group around the (x=-5, y=0) area contains 186 points representing 2.1% of the data, although outliers still show a continuous flow between morphology classes. From this visual inspection, it is clear that the fingerprints capture both drastic and gradual changes in structure.

Supplementary References

1. Nieves, D. J. *et al.* A framework for evaluating the performance of SMLM cluster analysis algorithms. *Nat. Methods* 20, 259–267 (2023).
2. Wallis, T. P. *et al.* Super-resolved trajectory-derived nanoclustering analysis using spatiotemporal indexing. *Nat. Commun.* 14, 3353 (2023).
3. Hou, Y. *et al.* Live-cell photoactivated localization microscopy correlates nanoscale ryanodine receptor configuration to calcium sparks in cardiomyocytes. *Nat. Cardiovasc. Res.* 2, 251–267 (2023).
4. Li, Y. *et al.* Real-time 3D single-molecule localization using experimental point spread functions. *Nat. Methods* 15, 367–369 (2018).
5. Zhang, M. *et al.* Direct observation of heterogeneous formation of amyloid spherulites in real-time by super-resolution microscopy. *Commun. Biol.* 5, 850 (2022).
